# Antibody RING-Mediated Destruction of Endogenous Proteins

**DOI:** 10.1016/j.molcel.2020.04.032

**Published:** 2020-07-02

**Authors:** Adel F.M. Ibrahim, Linnan Shen, Michael H. Tatham, David Dickerson, Alan R. Prescott, Naima Abidi, Dimitris P. Xirodimas, Ronald T. Hay

**Affiliations:** 1 Centre for Gene Regulation and Expression, School of Life Sciences, University of Dundee, Dundee DD1 5EH, UK; 2 Dundee Imaging Facility, School of Life Sciences, University of Dundee, Dundee DD1 5EH, UK; 3 Cell Biology Research Centre of Montpellier, CNRS, UMR 5237, Montpellier, France

**Keywords:** ARMeD, nanobody-RING fusion, ubiquitin, proteasome, E3 ligase, protein degradation

## Abstract

To understand gene function, the encoding DNA or mRNA transcript can be manipulated and the consequences observed. However, these approaches do not have a direct effect on the protein product of the gene, which is either permanently abrogated or depleted at a rate defined by the half-life of the protein. We therefore developed a single-component system that could induce the rapid degradation of the specific endogenous protein itself. A construct combining the RING domain of ubiquitin E3 ligase RNF4 with a protein-specific camelid nanobody mediates target destruction by the ubiquitin proteasome system, a process we describe as antibody RING-mediated destruction (ARMeD). The technique is highly specific because we observed no off-target protein destruction. Furthermore, bacterially produced nanobody-RING fusion proteins electroporated into cells induce degradation of target within minutes. With increasing availability of protein-specific nanobodies, this method will allow rapid and specific degradation of a wide range of endogenous proteins.

## Highlights

• Antibody RING-mediated destruction (ARMeD) targets endogenous proteins for degradation
• ARMeD is mediated by a nanobody fused to the RING domain of ubiquitin E3 ligase RNF4
• Nanobody-RING fusions introduced into cells degrade target proteins within minutes

## Graphical Abstract

**Figure:**
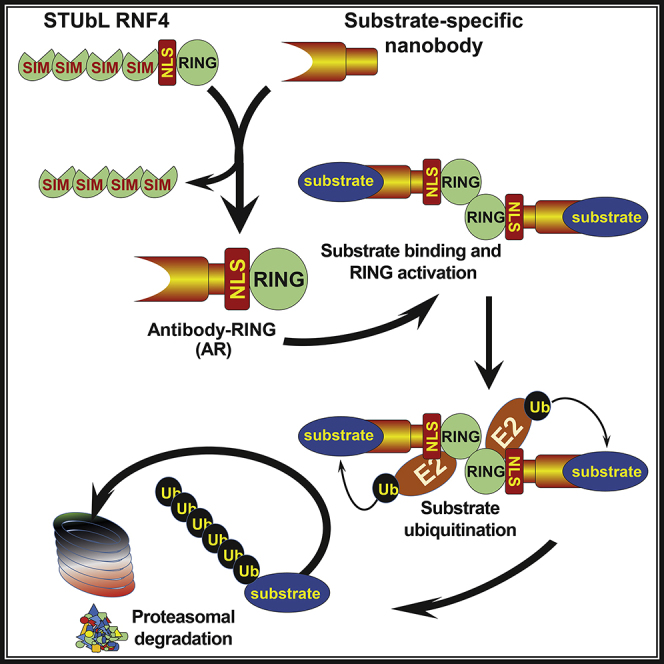

## Teaser

To study gene function, a single-component system that induces rapid degradation of the protein product of the gene was developed. Described as antibody RING-mediated destruction (ARMeD), a nanobody fused to the RING domain of ubiquitin E3 ligase RNF4 mediates degradation of the target protein by the ubiquitin proteasome system.

## Introduction

Understanding the function of a gene usually requires ablation of expression of the gene product. In traditional genetic and genome editing (Doudna and Charpentier, 2014) approaches, changes in genetic material result in inactivation, ablation of expression, or alteration of activity of the gene product that manifest themselves in an altered phenotype, presumed to be directly linked to the function of the protein. Alternative methods such as RNAi lead to destruction of the mRNA but have no direct effect on the protein product of the gene (Elbashir et al., 2001), which is thus depleted at a rate defined by the inherent half-life of the protein. This precludes the use of such approaches to remove proteins with a very long half-life (Toyama et al., 2013) or insoluble protein aggregates that are typically associated with neurological disease. RNAi-based approaches also have the disadvantage of taking a long time to deplete protein (typically 48 h), making it difficult to study processes like the cell cycle, where protein depletion is only achieved after multiple cell cycles. Such delays in protein depletion also give the cell time to initiate compensatory mechanisms that may mask the primary phenotype of target protein depletion. To directly induce degradation of a protein of interest, a number of approaches have harnessed the power and specificity of the protein degradation machinery of the cell. Proteins are first targeted for ubiquitination and then destroyed by the proteasome (Hershko and Ciechanover, 1998). Ubiquitin E3 ligases recognize substrates and mediate their ubiquitination. Most methods either artificially target the protein to a pre-existing ubiquitin E3 ligase or generate new E3 ligases engineered to recognize particular proteins. Proteolysis targeting chimeras (PROTACs) are bifunctional chemical entities that bind to target proteins and recruit them to a pre-existing ubiquitin E3 ligase (Bondeson et al., 2015, Runcie et al., 2016, Sakamoto et al., 2001). This approach has the advantage that endogenous proteins can be targeted, and the cell does not have to be modified. However, the protein to be targeted must bind with high affinity to a ligand contained within the PROTAC molecule, and such ligands do not exist for most proteins. A method that allows rapid, ligand-induced degradation of target proteins is the auxin inducible degron (AID) system (Holland et al., 2012, Nishimura et al., 2009). In this approach, non-plant cells are engineered to express the plant ubiquitin E3 ligase TIR1 that is inactive until it binds to the plant hormone auxin. In its active, auxin-bound state, it recognizes a specific protein sequence, known as a degron, which can be engineered into a protein to be targeted for degradation. In the absence of auxin, the protein is stable but undergoes rapid degradation when auxin is added to the medium of the cells. Although this approach enables rapid degradation of target protein, it necessitates engineering of cells to express plant TIR1 and a degron-tagged protein target. An alternative approach is to genetically modify a pre-existing ubiquitin E3 ligase to change its substrate specificity. This has been achieved by fusing the substrate recognition module of a cullin RING ligase (CRL) to a specific protein recognition domain such as a nanobody. In this way, GFP-tagged proteins have been targeted for degradation by a GFP binding nanobody fused to von Hippel-Lindau (VHL) E3 ligase (Caussinus et al., 2011, Fulcher et al., 2016). Most recently, “Trim-Away” has allowed acute and rapid destruction of endogenous proteins (Clift et al., 2017). This is a two-component system based on uptake of antibodies into cells where they recognize their target protein and also bind to the ubiquitin E3 ligase TRIM21 that mediates their ubiquitination and degradation. In cells that express TRIM21, only the antibody needs to be delivered to the cell to induce degradation, but in cells lacking TRIM21, both antibody and TRIM21 need to be expressed.

Although all of these methods have particular advantages and disadvantages, our objective was to develop a system based on a single component that could be produced in high yield and used as a reagent that could be introduced into cells and induce rapid and specific degradation of endogenous proteins. We thus fused the RING domain of ubiquitin E3 ligase RNF4 to camelid nanobodies that mediate destruction of the target of the nanobody by the ubiquitin proteasome system in a process we describe as antibody RING-mediated destruction (ARMeD). Proteomic analysis indicates that this approach is remarkably specific with no observable off-target effects. We also found that bacterially produced nanobody-RING fusion proteins electroporated into cells induce degradation of endogenous target proteins within minutes. We expect this technology will become increasingly adopted as availability of protein-specific nanobodies increases.

## Results

### ARMeD

**Figure 1:**
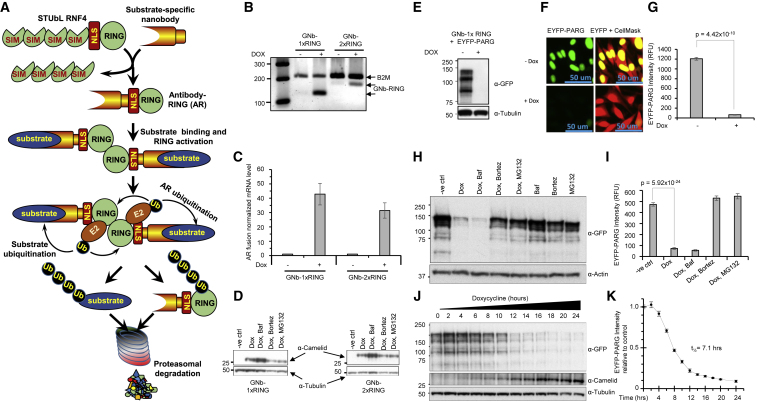
Antibody-RING Mediated Destruction (ARMeD)—Principle, Tool Development, and Degradation of EYFP-PARG (A) Schematic representation of the principle of ARMeD. SUMO recognition motifs (SIMs) of the SUMO-targeted ubiquitin ligase (STUbL) RNF4 are replaced with a nanobody targeting a protein substrate of interest. Expression of this fusion protein allows binding of substrate to nanobody and RING-mediated ubiquitination leading to proteasomal degradation. HeLa Flp-in/T.Rex cells engineered to inducibly express GNb-1xRING or GNb-2xRING were either untreated (−) or doxycycline-treated (+) for 24 h. (B) mRNA levels were analyzed by qRT-PCR with beta-2 microglobulin (B2M) as housekeeping control, and products at 24 cycles were separated on an agarose gel. (C) Quantitative expression data were obtained from three independent RNA preparations from each condition, normalized to B2M mRNA and uninduced control samples. Error bars represent mean ± SD from three independent replicates. (D) Protein levels were analyzed by western blotting using an anti-camelid antibody. (E and F) HeLa Flp-in/T.Rex cells engineered to inducibly express GNb-1xRING and stably express YFP-PARG were induced as above and analyzed by western blotting using an anti-GFP antibody (E), or cells were grown in 96-well plates fixed and visualized by high-content (HC) imaging using IN Cell analyzer 2000 (F). (G) HC data were obtained from 152,668 (uninduced) or 80,745 (induced) cells in 6 wells, and quantitation of intracellular YFP performed using the InCell Developer toolbox. YFP intensity data are plotted as the mean of 6 wells ± SD. (H and I) To establish the pathway of protein degradation, cells were incubated with autophagy inhibitor bafilomycin A1 (Baf, 100 nM) or proteasome inhibitors bortezomib (1 μM) or MG132 (10 μg/mL) for 1.5 h prior to 16 h doxycycline induction. The role of other E3 ligases in degradation of substrate was examined by subjecting cells to inhibitors without Dox induction. Western blotting (H) and HC analysis (I) were performed as above. HC YFP-PARG intensity data were obtained from 20,000–40,000 cells grown in 12 wells of a 96-well plate for each condition and plotted as the mean of 12 well replicates ± SD (I). (J and K) YFP-PARG degradation and production of the ARMeD fusion was assessed in a time course experiment by collecting cells at indicated times after doxycycline addition. Samples were analyzed by western blotting (J) or HC imaging (K). HC YFP intensity data were obtained from 115,000–250,000 cells grown in 8 wells of a 96-well plate for each time point, normalized to uninduced control cells, and plotted as the mean of 8 well replicates ± SD. Statistical analysis was performed by a two-tailed unpaired t test.

Ubiquitin E3 ligase RNF4 contains a C-terminal RING domain responsible for dimerization and recruitment of the ubiquitin loaded E2 conjugating enzyme, whereas the N-terminal region contains 4 SUMO interaction motifs (SIMs) that allow the E3 ligase to engage substrates containing multiple SUMOs (Figure 1A). When SUMO modified substrate is bound and ubiquitin-loaded, E2 is primed for catalysis (Dou et al., 2012, Plechanovová et al., 2012, Pruneda et al., 2012) a nucleophile (usually the ε-amino group of lysine) attacks the thioester bond linking ubiquitin to the active site of the E2 and ubiquitin is transferred to substrate (Figure 1A). To allow the E3 ligase to be used against any defined target, we sought to change the substrate recognition properties of RNF4. The SUMO recognition domain was therefore replaced with a camelid nanobody that could direct the RING domain of RNF4 (nanobody-1xRING) to the target of the nanobody. We also generated a constitutively dimeric form of RNF4 (nanobody-2xRING) by linking the nanobody to two copies of RNF4 RING connected by a short linker (Branigan et al., 2015, Plechanovová et al., 2012, Rojas-Fernandez et al., 2014). The nuclear localization signal (NLS) of RNF4 was retained in all constructs to allow efficient targeting of nuclear proteins. Initially, we used a well-characterized nanobody raised against GFP that also recognizes yellow fluorescent protein (YFP) (Kirchhofer et al., 2010). These constructs were used to generate HeLa Flp-in/T Rex cells where expression of the GFP-nanobody RING fusions was doxycycline (Dox)-dependent (Figures 1B–1D). Our expectation was that expression of a nanobody RING fusion in cells would lead to ubiquitin-proteasome mediated degradation of target protein. We describe this process as ARMeD. To test this hypothesis, we stably expressed poly ADP ribose glycohydrolase (YFP-PARG) in the HeLa Flp-in/T Rex cells already expressing the Dox-inducible GFP-nanobody RING fusions. Western blotting indicated that after Dox induction of GFP-nanobody RING (GNb-1xRING), YFP-PARG was no longer detectable by western blotting (Figure 1E). Fluorescence imaging also revealed that Dox induction led to depletion of PARG in almost all cells (Figure 1F), whereas high content image analysis revealed that Dox induction led to a 19-fold reduction in YFP-PARG levels (Figure 1G). To distinguish between the two main modes of ubiquitin-dependent degradation, Dox induction was carried out in the presence of autophagy inhibitor bafilomycin A1 or proteasome inhibitors MG132 or bortezomib. Western blotting (Figure 1H) and high content imaging (Figure 1I) indicated that GNb-1xRING induced degradation of YFP-PARG was unaffected by bafilomycin, but was blocked by both MG132 and bortezomib. Thus, GFP-nanobody RING induced degradation of YFP-PARG via the ubiquitin proteasome system. To establish the time course of degradation, GNb-1xRING was induced by Dox and YFP-PARG expression monitored by western blotting (Figure 1J) and high content imaging (Figure 1K) over a 24-h period. The t_1/2_ determined from the quantitative imaging data was 7.1 h. To a large extent, this represents the time taken for the GNb-1xRING to accumulate to levels sufficient to induce RING dimerization and E3 ligase activity (Figure 1J).

**Figure 2:**
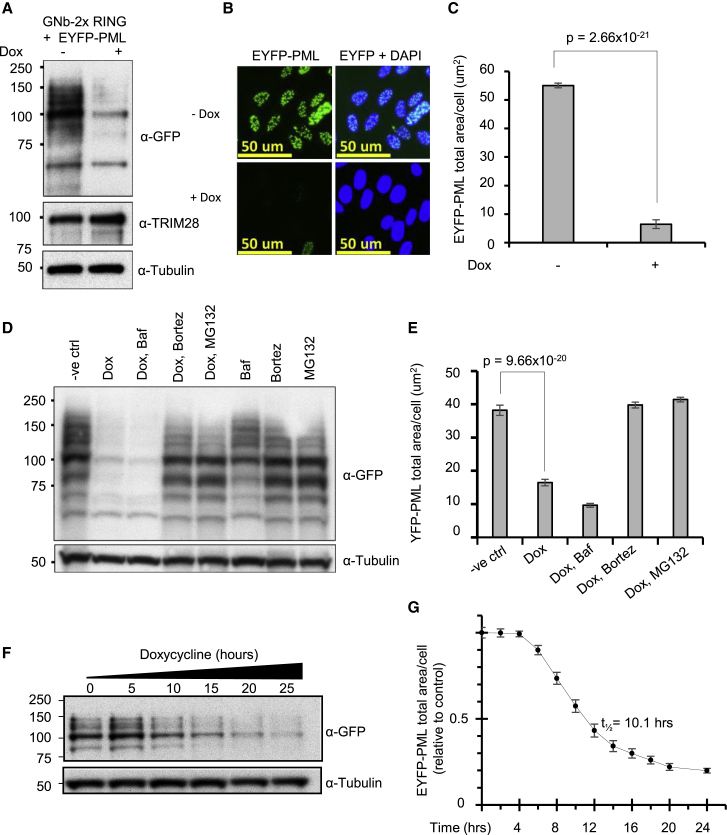
ARMeD of YFP-PML (A and B) HeLa Flp-in/T.Rex cells engineered to inducibly express GNb-2xRING and stably express YFP-PML were either untreated (−) or Doxycycline treated (+) for 24 h. Protein levels were analyzed by western blotting using an anti-GFP antibody (A) or analyzed by HC imaging using IN Cell analyzer 2000 (B). (C) HC data were obtained from 33,775 (uninduced) or 33,434 (induced) cells in 9 wells, and quantification of YFP fluorescence was performed using the InCell Developer toolbox. Data representing YFP-PML total area/cell are plotted as means of 9 wells ± SD. (D and E) To establish the pathway of protein degradation, cells were incubated with autophagy inhibitor bafilomycin A1 (Baf, 100 nM) or proteasome inhibitors bortezomib (1 μM) or MG132 (10 μg/mL) for 1.5 h prior to 16 h doxycycline induction. The role of E3 ligases other than the ARMeD fusion in degradation of substrate was examined by subjecting cells to inhibitors without Dox induction. Western blotting (D) and HC analysis (E) were performed as above. The HC YFP-PML data (total area/cell) were obtained from 20,000–50,000 cells grown in 12 wells of a 96-well plate for each condition and plotted as the mean of 12 well replicates ± SD (E). (F and G) YFP-PML degradation was assessed over time by collecting cells at indicated times after doxycycline addition and performing western blotting (F) or high-content analysis (G). The HC EYFP-PML data (total area/cell) were obtained from 20,000–25,000 cells grown in 8 wells of a 96-well plate for each time point, normalized to uninduced control cells, and plotted as the mean of 8 well replicates ± SD. Statistical analysis was performed by a two-tailed unpaired t test.

Although YFP-PARG is a soluble nuclear protein, a more demanding test of the utility of the GFP-nanobody RING was its ability to induce degradation of YFP-PML (promyelocytic leukemia) protein that is located in nuclear bodies and is stabilized in these bodies by a dense network of SUMO-SIM interactions (Shen et al., 2006). Cells expressing YFP-PML and a Dox inducible GFP-nanobody 2xRING (GNb-2xRING) were generated. Western blotting indicated that after Dox induction of GNb-2xRING, YFP-PML levels were dramatically reduced (Figure 2A). However, the PML body component TRIM28, which interacts with PML via SUMO, was unaffected (Figure 2A), indicating that ARMeD is highly specific for the protein targeted by the nanobody. Fluorescence imaging also revealed that Dox induction led to depletion of PML in almost all cells (Figure 2B) while high content analysis of the images revealed that Dox induction led to a 9 fold reduction in YFP-PML levels (Figure 2C). Western blotting (Figure 2D) and high content imaging (Figure 2E) indicated that although GNb-2xRING induced degradation of YFP-PML was not inhibited by bafilomycin, it was blocked by both MG132 and bortezomib. Thus, GNb-2xRING also induces degradation of YFP-PML via the ubiquitin proteasome system. Time course analysis of the degradation of YFP-PML over a 25-h period monitored by western blotting (Figure 2F) or over a 24-h period analyzed by high content imaging (Figure 2G) showed the t_1/2_ to be slightly longer than for YFP-PARG at 10.1 h. However, we note that the degradation curves for YFP PARG and YFP-PML were an imperfect fit to the exponential equations used that could be due to the delayed onset of degradation and, therefore, the actual t_1/2_ may be even shorter than the one calculated on the basis of this equation for both proteins.

**Figure 3:**
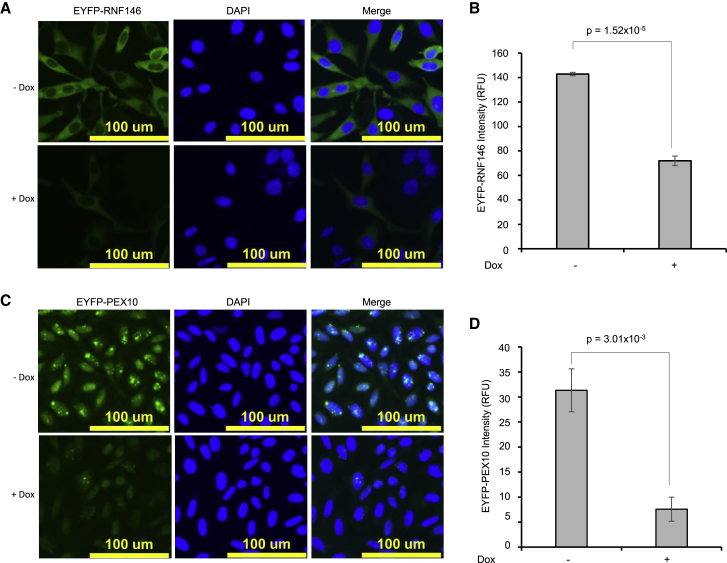
ARMeD of YFP-RNF146 and YFP-PEX10 (A and C) HeLa Flp-in/T.Rex cells engineered to inducibly express GNb-1xRING and stably express YFP-RNF146 (A) or PEX10 (C) were grown in 96-well plates and either untreated (−Dox) or doxycycline-treated (+Dox) for 24 h, fixed, and visualized by HC imaging using IN Cell analyzer 2000. (B and D) HC data were obtained from 45,278 (uninduced) or 45,749 (induced) cells for RNF146 (B) or from 128,710 (uninduced) or 110,743 (induced) cells for PEX10 (D), and quantitation of intracellular YFP was performed using InCell Developer toolbox. YFP intensity data are plotted as mean of 3 wells ± SD. Statistical analysis was performed by a two-tailed unpaired t test.

Although targeting the above two proteins was likely to succeed due to the presence of the functional nuclear localization signal (NLS) of RNF4, we also proposed that the nanobody-RING fusions might also be active while on transit from their cytoplasmic synthesis site to the nucleus. To test this hypothesis, we generated HeLa Flp-in/T Rex cells expressing Dox-inducible GFP-nanobody 1xRING (GNb-1xRING) along with YFP-tagged E3 ubiquitin-protein ligase RNF146 or YFP-tagged peroxisomal biogenesis factor 10 (PEX10). PEX10 is an integral membrane protein of the peroxisome where both its N and C termini project into the cytoplasm (Okumoto et al., 1998). Doxycycline induction of the GNb-1xRING fusion in those cell lines led to a 2-fold reduction of YFP-tagged RNF146 and a 5-fold reduction of YFP-PEX10 (Figure 3). We conclude that our GFP-nanobody-RING fusions can be used for targeting both nuclear and cytosolic proteins.

### ARMeD of Endogenous NEDD8-Specific Protease NEDP1

**Figure 4:**
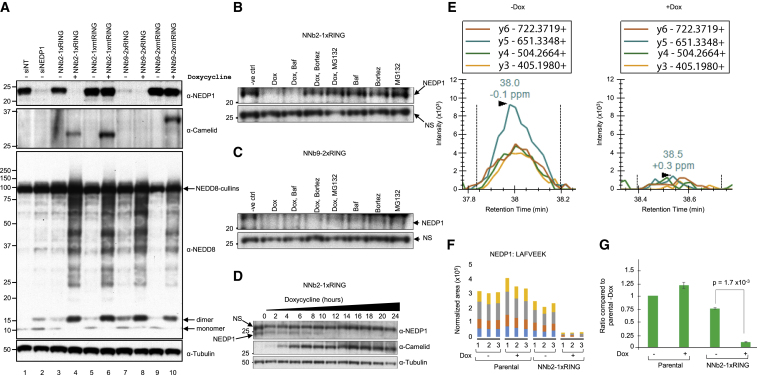
Degradation of Endogenous NEDD8 Protease NEDP1 with ARMeD Constructs (A) HeLa Flp-in/T.Rex cells were transfected with non-targeting (siNT, lane 1) or NEDP1 (siNEDP1, lane 2) siRNA, and cell extracts harvested 72 h after transfection. Lanes 3–10: HeLa Flp-in/T.Rex cells engineered to inducibly express NEDP1 specific nanobody-RING constructs were untreated (−) or doxycycline-treated (+) for 24 h. Protein levels were analyzed by western blotting using anti-NEDP1, anti-camelid, and anti-NEDD8 antibodies. α-Tubulin was used as loading control. NEDD8-cullins and NEDD8 monomers and dimers are indicated by arrows. (B and C) To establish the pathway of protein degradation by NNb2-1xRING (B) and NNb9-2xRING (C), cells were incubated with autophagy inhibitor bafilomycin A1 (Baf, 100 nM) or proteasome inhibitors bortezomib (1 μM) or MG132 (10 μg/mL) for 1.5 h prior to 16 h doxycycline induction. The role of other E3 ligases in degradation of substrate was examined by subjecting cells to inhibitors without Dox induction. (D) Induction of NEDP1 ARMeD fusions and rate of NEDP1 degradation after doxycycline addition was assessed by western blotting using anti-NEDP1 and anti-camelid antibodies. A non-specific (NS) band recognized by the NEDP1 antibody served as an additional loading control. (E) Parallel reaction monitoring to quantify NEDP1 depletion. Example MS2 chromatograms for fragment ions y3–y6 of the NEDP1 peptide LAFVEEK with and without doxycycline treatment. Dashed lines are peak boundaries as reported by Skyline. Mass errors for most prominent peaks are labeled as ppm. (F) Example MS2 peak areas used for quantification of NEDP1 ARMeD knockdown (NNb2-1xRING) and control strain (parental). (G) Median enrichment or depletion compared to PARENTAL-dox of the LAFVEEK, LEAFLGR, and QVAEKLEAFLGR peptides. Error bars are SE. Statistical analysis was performed by a two-tailed unpaired t test. See also Figure S1 and Table S2.

To explore the application of ARMeD to endogenous, unmodified proteins it was necessary to generate protein-specific nanobodies. The NEDD8 specific protease NEDP1 (Mendoza et al., 2003), has been structurally resolved (Shen et al., 2005) and the consequences of its depletion previously established (Bailly et al., 2019). We generated a series of camelid nanobodies against NEDP1, among which nanobody 2 and nanobody 9 both bind to NEDP1 and inhibit its catalytic activity (to be described in detail elsewhere). To test their activity *in vivo* nanobody 2 was fused to single RING of RNF4 (NNb2-1xRING) while nanobody 9 was fused to a constitutively dimeric form of RNF4 (NNb9-2xRING). Nanobody 2 was also fused to single RING of RNF4 inactivated by the double mutation M140A, R181A (Plechanovová et al., 2011) (NNb2-1xmtRING) while nanobody 9 was fused to a similarly mutated constitutively dimeric form of RNF4 (NNb9-2xmtRING). The mutated residues correspond to M136 and R177 in human RNF4 but the RING domain sequence is identical in both orthologs. These constructs were used to generate HeLa Flp-in/T Rex cells where expression of the NEDP1-nanobody RING fusions was Dox-dependent. Expression of the fusions was induced by Dox treatment for 24 h, while cells treated with a pool of small interfering RNAs (siRNAs) to NEDP1 or non-targeting controls for 48 h were used for comparison. Analysis by western blotting revealed that after Dox treatment NNb2-1xRING, but not its inactive mutant counterpart, induced the degradation of NEDP1to undetectable levels (Figure 4A). In comparison, siRNA reduced the level of NEDP1, but depletion was incomplete and NEDP1 could still be detected. Even before application of Dox, NEDP1 levels were reduced in cells containing the NNb9-2xRING construct. After Dox treatment NEDP1 levels were reduced to undetectable levels. Again, mutational inactivation of the RING blocked NEDP1 degradation. In all situations, apart from NNb9-2xRING, Dox induction resulted in the accumulation of the nanobody-RING fusions at the correct molecular weight. In the case of NNb9-2xRING, NEDP1 degradation is apparent even in the absence of Dox. This is due to leaky, Dox-independent expression as determined by RT-PCR (Figures S1A and S1B). As the fused RINGs create a hyperactive E3 ligase, even the small amount produced under these conditions results in substantial NEDP1 depletion. After Dox induction, NEDP1 is undetectable by western blotting but the NNb9-2xRING fusion is also undetectable (Figure 4A). This is likely due to auto-ubiquitination of the E3 ligase as the mutated, inactive form is detected, and mRNA encoding NNb9-2xRING is induced by Dox (Figure S1B).

NEDP1 depletion with NNb2-1xRING or NNb9-2xRING leads to the accumulation of NEDD8 conjugates and the appearance of NEDD8 dimers (Figure 4A). When NEDP1 is depleted with siRNA NEDD8 dimers and higher molecular weight conjugates are only modestly increased. Counterintuitively, although NEDP1 is not depleted, expression of NNb2-1xmtRING NNb9-2xmtRING leads to accumulation of NEDD8 modified species (Figure 4A). This is explained by the direct inhibition of the activity of NEDP1 by the nanobodies, even though NEDP1 is not turned over.

To determine the mode of NEDP1 degradation by NNb2-1xRING and NNb9-2xRING, Dox induction was carried out in the presence of the autophagy inhibitor bafilomycin A1, or proteasome inhibitors MG132 or bortezomib. Western blotting indicated that NNb2-1xRING-induced degradation of NEDP1 was unaffected by bafilomycin, but was blocked by both MG132 and bortezomib (Figure 4B). This was less evident with the hyperactive constitutively dimeric NNb9-2xRING, because proteasome inhibitors had only limited ability to block NEDP1 degradation (Figure 4C). Thus, the NNb-RING fusions appeared to induce degradation of NEDP1 via the ubiquitin proteasome system. To establish the time course of degradation NNb-1xRING was induced by Dox, and NEDP1 expression was monitored by western blotting (Figure 4D) over a 24-h period. Degradation of NEDP1 is evident at early times, but is complete by 12 h (Figure 4D), a time when the NNb-1xRING has accumulated to levels (Figure 4D) that are probably high enough to induce RING dimerization and E3 ligase activity.

Although NNb2-1xRING and NNb9-2xRING reduce NEDP1 to undetectable levels by western blot, we used the highly sensitive mass spectrometry technique parallel reaction monitoring (PRM) (Peterson et al., 2012) to obtain quantitative analysis of the scale of NEDP1 depletion. Three well resolved peptides from NEDP1 were selected for analysis, and for each peptide a number of fragment ions were quantified (Figure 4E). Combining the data for the three peptides indicates that NNb2-1xRING reduces NEDP1 levels by at least 8-fold (Figures 4F and 4G), and some NEDP1 peptide fragments become undetectable even by this method on Dox treatment and therefore cannot contribute to the final calculations (Figure 4E).

### Selectivity of ARMeD

**Figure 5:**
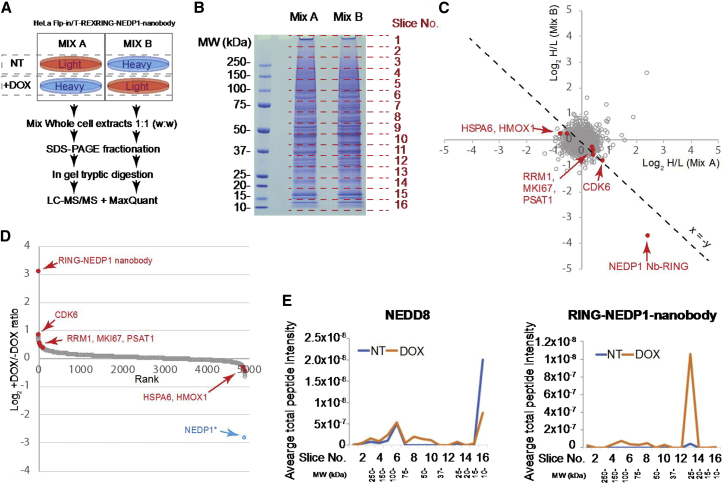
Total Proteome Consequences of Nanobody-RING Fusion Expression (A) Design of a SILAC experiment to identify protein abundance changes to cells during induction of the NEDP1 nanobody-RING by Dox. (B) Gel image of whole cell extracts from SILAC mixes as shown from (A). (C) Scatterplot showing the SILAC H/L ratio data for 4,907 proteins common to data derived from two SILAC mixes. Grey markers indicate proteins not identified as significantly different in both comparisons nor consistently responding to Dox. Red markers are proteins with significant abundance change after Dox treatment that is consistent across both mixes. x = -y line shown to highlight consistently responding proteins. (D) Rank versus average response to Dox across both SILAC mixes for the 4,907 proteins shown in (C) CDK6, cyclin-dependent kinase 6; RRM1, ribonucleoside-diphosphate reductase large subunit; MKI67, marker of proliferation Ki-67; PSAT1, phosphoserine aminotransferase 1; HSPA6, heat shock protein family A (Hsp70) member 6; HMOX1, heme oxygenase 1. NEDP1^∗^ included for comparison; NEDP1 data derived from PRM experiment shown in Figure 4 and not from this SILAC experiment. (E) Slice-specific total protein intensity data for NEDD8 (left panel) and the NEDP1 (right panel) nanobody-RING construct. For each slice, the average intensity across both mixes is shown. See also Figure S2 and Table S1.

The target specificity of the ARMeD approach was evaluated by shotgun proteomic analysis of crude cell lysates from cells containing the Dox-inducible NEDP1 nanobody fused RING (NNb2-1xRING). A stable isotope labeling by amino acids in cell culture (SILAC) (Mann, 2006) approach was taken whereby cells treated with vehicle only or Dox induced were grown in “Light” or “Heavy” SILAC medium to allow comparison between the two in both “forward” and “reverse,” label-swapped formats (mixes A and B in Figure 5A). Whole cell extracts were prepared, mixed in a 1:1 ratio, and fractionated by SDS-PAGE (Figure 5B). Each lane was cut into 16 slices, each slice was subjected to in-gel trypsin digestion, and peptides were analyzed by mass spectrometry. The data from both mixes were analyzed in MaxQuant, and the Log_2_ H/L ratios for all common identifications are displayed on a scatterplot (Figure 5C). Of the 4,907 proteins detected in all 4 SILAC conditions, the only protein to show a consistent change above 2-fold after Dox induction was the NNb2-1xRING fusion protein (Figures 5C–5E). NEDP1 itself was not detected in this study even though the dynamic range covered proteins from a little as 100 copies per cell and covered a similar range as other studies of a similar scale (Figure S2). The previously described PRM approach had determined that Dox induction reduced its level by 8-fold (Figures 4E–4G). Because NEDP1 depletion leads to an accumulation of NEDD8 conjugates (Figure 4A), we analyzed the distribution of NEDD8 peptides in each of the gel slices under each condition. This revealed that Dox induction led to a decrease in the intensity of NEDD8 peptides in the region of the gel containing unconjugated NEDD8 (slice 16) and a general increase in the intensity of NEDD8 peptides in some higher MW regions (slices 7–11) (Figure 5E). However, the region of the gel containing NEDD8 modified cullins (slice 6) was unaffected after NEDP1 depletion (Figure 5E). Furthermore, the NNb2-1xRING construct itself also displayed higher molecular weight forms on induction (Figure 5F), consistent with a mechanism of self-ubiquitination as described above. Thus, the nanobody-directed RING fusion displays remarkable specificity for its target protein, and although we did not detect off-target degradation with NNb2-1xRING, we cannot be sure that this does not take place for proteins that we do not detect. It is also likely that off-target effects will vary between nanobodies.

### Acute and Rapid Degradation of Target Proteins by Purified Nanobody-RING Fusions

**Figure 6:**
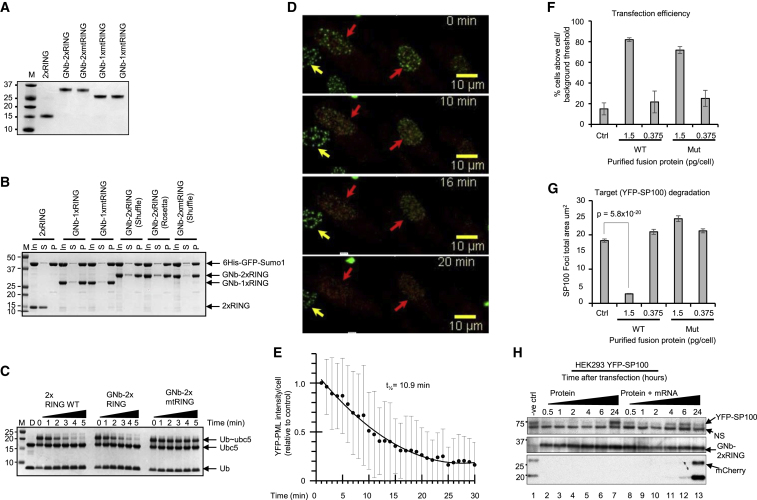
Acute and Rapid Degradation of Target Proteins by ARMeD Proteins (A) Coomassie-blue stained SDS-PAGE analysis of purified GFP nanobody-RING fusions, wild-type (WT) (GNb-1xRING and GNb-2xRING), and (GNb-1xmtRING and GNb-2xmtRING). (B) Nickel bead pull-down assays of recombinant 6His-GFP-SUMO1 with nanobody-RING fusions evaluated with SDS-PAGE and Coomassie staining (I, input; S, supernatant; P, pull-down). Fused RNF4 RING (2xRING) is used as negative control. Shuffle and Rosetta are bacterial strains used for expressing recombinant fusion proteins. (C) Lysine discharge assays with ubiquitin loaded Ubc5 (Ub-Ubc5) in presence of fused RNF4 RING (2xRING), GNb-2xRING, and GNb-2xmtRING. Samples were removed at indicated times (minutes) and analyzed by non-reducing SDS-PAGE. A sample reduced with DTT is indicated. (D) HeLa Flp-in/T.Rex cells stably expressing YFP-PMLIII were injected with a 1:1 mixture of GNb-2xRING and mCherry-SIM, and images were collected every 2 min. Injected cells (red arrow) and uninjected cells (yellow arrow) are indicated. The images shown were taken at 0, 10, 16, and 20 min following injection. (E) Injected cells were identified using mCherry fluorescence, and mean summed YFP intensity of injected cells was obtained following background subtraction and plotted for each time point ± SD. (F and G) Efficiency of (F) purified protein delivery to cells and (G) target protein degradation. HEK293 cells stably expressing YFP-SP100 were electroporated with a mix of mCherry-SIM protein and either GNb-2xRING or GNb-2xmtRING (either 0.375 pg or 1.5 pg of each purified protein/cell) and (F) mCherry or (G) YFP fluorescence analyzed by HC imaging using IN Cell analyzer 2000. HC data were obtained from 29,923/26,007 (0.375/1.5 pg GNb-2xRING/cell) or 21,901/32,866 (0.375/1.5 pg GNb-2xmtRING/cell) cells in 12 wells, and quantitation of each fluorescence signal was determined individually using InCell Developer toolbox. Percentage of cells with mCherry fluorescence above background are plotted as means of 12 wells ± SD (F); and YFP fluorescence representing PML/SP100 foci total area/cell are plotted as means of 12 wells ± SD (G). Statistical analysis was performed by a two-tailed unpaired t test. (H) Rate of degradation was assessed by electroporating HEK293 YFP-SP100 cells above with 12 μg of purified GNb-2xRING in a total volume of 100 μL and collecting samples at the indicated times after electroporation (lanes 2–7). To prolong the activity of the GNb-2xRING fusion, the cells were co-transfected with the same amount of the purified recombinant fusion and 500 fg/cell of an *in vitro*-generated GNb-2xRING mRNA combined with a similar amount of similarly generated mCherry mRNA as a transfection internal positive control (lanes 8–13). Samples were collected at the same time intervals as the GNb-2xRING fusion without mRNA transfections (lanes 2–7). Negative control cells (lane 1) were only transfected with 1 pg/cell of the mCherry mRNA and collected after 6 h. YFP-SP100 degradation was scored by western blotting, using an anti-GFP antibody. A non-specific band (NS) just below the target protein served as a loading control. GNb-2xRING was detected using an anti-camelid antibody and expression of electroporated mRNAs was monitored using an anti-mCherry antibody. See also Figures S3 and S4 and Videos S1 and S2.

Although much faster than indirect nucleic acid-based methods for protein manipulation, Dox-induced nanobody-RING fusions act over a timescale of hours. This will include time taken for the chemical to be absorbed into cells and the construct itself to be expressed in quantities required to degrade the target (Figures 1J and 4D). Furthermore, the Dox-inducible system also requires genetic manipulation of the cell population. To circumvent these issues and to attempt to hasten protein degradation, we decided to directly introduce purified nanobody-RING fusion proteins into cells. Thus GNb-1xRING, GNb-2xRING, and their inactive RING counterparts (containing the M140A, R181A double mutation) GNb-1xmtRING and GNb-2xmtRING, were expressed in bacteria and purified to homogeneity (Figure 6A). To confirm that the purified proteins retained target binding and E3 ligase activity, we carried out *in vitro* experiments. GNb-1xRING, GNb-2xRING, GNb-1xmtRING, and GNb-2xmtRING, but not an RNF4 fused RING alone, efficiently pulled down a 6His-GFP-SUMO1 fusion protein (Figure 6B). Ubiquitin E3 ligase activity was tested in a substrate-independent fashion using a lysine discharge assay (Branigan et al., 2015) that measures the ability of the RING to activate the ubiquitin-Ubc5 thioester bond to nucleophilic attack by free lysine. The RNF4-fused RING alone and the GNb-2xRING, but not the GNb-2xmtRING, were active in lysine discharge activity (Figure 6C). To test the activity of the nanobody-RING fusion proteins *in vivo*, we used microinjection to introduce purified GNb-2xRING into cells expressing YPF-PML. Microinjected cells were marked by co-injection of an mCherry protein (Figure 6D), and the fluorescent images were collected in real time (Video S1; still of Video S1 shown in Figure S3). Quantitation of the YFP signal from PML revealed that the protein was degraded with a t_1/2_ of 10.9 min (Figure 6E). Although microinjection demonstrates the principle that purified GNb-2xRING can be used as a single component reagent to induce target protein degradation, we sought to extend this to rapid, time-resolved degradation in a bulk populations of cells. A variety of methods were therefore tested for the simultaneous delivery of GNb-2xRING to a large numbers of cells. As a transfection efficiency control, mCherry was included with GNb-2xRING. Neon electroporation proved to be the most satisfactory approach. Using high content imaging, we could demonstrate that 1.5 pg of electroporated protein/cell resulted in a high proportion (>80%) of cells displaying mCherry fluorescence above background levels (Figure 6F). To assess target degradation, GNb-2xRING was electroporated into cells expressing the PML body component SP100 as a YFP fusion protein. High content imaging was used to evaluate the extent of degradation of YFP-SP100 after 60 min. Using only 0.375 pg of GNb-2xRING/cell, little degradation was observed, but with 1.5 pg of GNb-2xRING/cell, SP100 levels were reduced by 85% (Figure 6G). The time taken for degradation of YFP-SP100 was determined by electroporating cells with purified GNb-2xRING and cells processed for high content imaging at various times post-electroporation. High content imaging indicates that maximal degradation has been reached 10 min after electroporation (Figure S3B).

Video S1 ARMeD of YFP-PMLIII in HeLa Cells, Related to Figure 6

To determine the duration of degradation, cells were either electroporated with purified GNb-2xRING protein or electroporated with a combination of purified GNb-2xRING protein and capped and polyadenylated mRNA encoding GNb-2xRING. It is clear that degradation of YFP-SP100 is sustained for 6 h after electroporation with purified protein, but levels of YFP-SP100 return to normal after 24 h (Figure 6H). However, with the combination of purified protein and mRNA, degradation is sustained for 24 h (Figure 6H). In practical terms, this means that purified protein is ideal for short term elimination of the protein, but if this needs to be maintained, this can be done by the inclusion of mRNA. Thus, purified preparations of nanobody-RING fusions can be used as a reagent to rapidly degrade target proteins in bulk populations of cells. As a representative of a cytoplasmic, cytoskeletal protein, we tested GFP-tubulin and demonstrated by western blotting and immunofluorescence that it was rapidly degraded after electroporation of bacterially expressed GNb-2xRING (Figure S4; Video S2).

Video S2 ARMeD of GFP-Tubulin in MCF7 Cells, Related to Figure 6

### Rapid Antibody-RING-Mediated Destruction of Endogenous, Unmodified NEDP1

**Figure 7:**
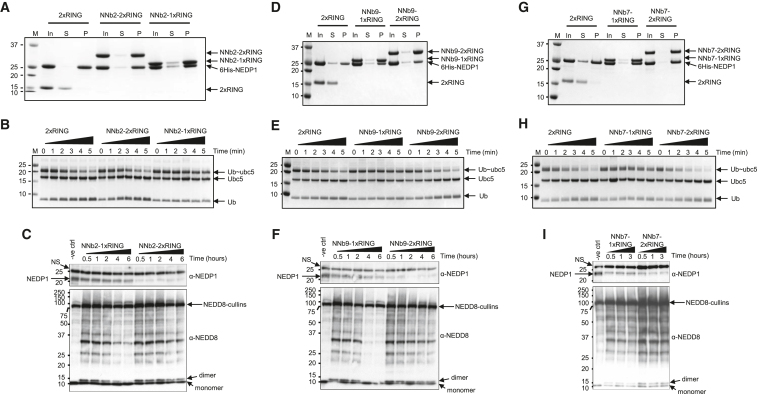
Rapid Antibody RING-Mediated Destruction of Endogenous NEDP1 (A, D, and G) Nickel bead pull-down assays of recombinant 6His-NEDP1 with nanobody-RING fusions NNb2-1xRING and 2xRING (A), NNb9-1xRING and 2xRING (D), and NNb7-1xRING and 2xRING (G) were evaluated with SDS-PAGE and Coomassie staining (In, input; S, supernatant; P, pull-down). Fused RNF4 RING (2xRING) is used as negative control. (B, E, and H) The activity of the NNb2-1xRING and 2xRING (B), NNb9-1xRING and 2xRING (E), and NNb7-1xRING and 2xRING (H) was tested in lysine discharge assays with ubiquitin loaded Ubc5 (Ub-Ubc5). A fused RNF4 RING (2xRING) served as a positive control. Samples were removed at indicated times (minutes) and analyzed by non-reducing SDS-PAGE. (C, F, and I) To assess activity of purified recombinant NEDP1 ARMeD fusions in cells, HEK293 cells were electroporated with NNb2-1xRING and 2xRING (C), NNb9-1xRING and 2xRING (F), or NNb7-1xRING and 2xRING (I) and harvested at the indicated time point after electroporation. NEDP1 and NEDD8 were analyzed by western blotting. NEDP1, a non-specific band (NS), NEDD8-cullins, and NEDD8 monomers and dimers are indicated by arrows. See also Figures S5 and S6.

Although we have established that purified GNb-2xRING can induce rapid degradation of a YFP-modified protein in a large population of cells, the ultimate test of ARMeD is the demonstration that it can induce the rapid degradation of endogenous, unmodified protein targets. We therefore evaluated the ability of the NEDP1 protease-specific nanobody-RING fusions to induce the rapid degradation of NEDP1 in bulk populations of HEK293 cells. Three different nanobodies (NNb2, NNb7, and NNb9) against NEDP1 were fused to 1xRING and 2xRING, expressed in bacteria, and purified to homogeneity as indicated (Figure S5). To confirm that the purified proteins retained their biological activities of binding to NEDP1 and E3 ligase activity, *in vitro* experiments were conducted. NNb2-, NNb7-, and NNb9-1xRING and 2xRING fusions, but not an RNF4 fused RING alone, efficiently pulled down a 6His-NEDP1 protein (Figures 7A, 7D, and 7G). Although binding of the NNb2 and NNb9-RING fusions to NEDP1 lead to loss of NEDD8 processing activity, binding of the NNb7-RING fusions did not lead to loss of processing activity (Figure S6B). Ubiquitin E3 ligase activity was tested in a lysine discharge assay as described above. The RNF4 fused RING alone and NNb2-, NNb7-, and NNb9-2xRING fusions, displayed comparable E3 ligase activity but the NNb2-, NNb7-, and NNb9-1xRING fusions were less active. (Figures 7B, 7E, and 7H). The ability to degrade endogenous, unmodified NEDP1 was determined by electroporating cells with purified NNb2-, NNb7-, and NNb9-1xRING and 2xRING fusions and cells collected at various times post-electroporation. Western blotting indicates that the 2xRING fusions efficiently induce the degradation of NEDP1 by 30 min, whereas degradation induced by the 1xRING fusions was less efficient (Figures 7C, 7F, and 7I). The phenotypic output of cells depleted for NEDP1 is the appearance of NEDD8 dimers and the accumulation of NEDD8 modified proteins. NNb2-, NNb7-, and NNb9-1xRING and 2xRING fusions all induce the appearance of NEDD8 dimers and the accumulation of higher molecular weight NEDD8-modified species, irrespective of their ability to inhibit NEDP1 processing activity (Figures 7C, 7F, and7I). Thus, with the appropriate nanobody-RING fusion, unmodified, endogenous cellular proteins can be rapidly targeted for degradation using purified preparations of the nanobody-RING fusion in bulk populations of cells.

## Discussion

Conventional gene knockouts and RNAi are widely used approaches to analyze the biological function of proteins. However, the disadvantages of these approaches are that it takes a long time (days) to effectively ablate mRNA expression, and once translation of the target protein has ceased, depletion of the protein is entirely dependent on its inherent half-life. In fact, many components of essential cellular structures, such as the nuclear pore complex, are stable over many months (Toyama et al., 2013) and would thus be resistant to depletion using methods that target RNA or DNA. Likewise protein aggregates that are the hallmark of neurological disease are stable over years. Here, we describe ARMeD as a route to circumventing these problems. In this approach, the RING domain of RNF4 is fused to a nanobody to create a small ubiquitin E3 ligase with unique target specificity that can be used to target the protein recognized by the nanobody for ubiquitin proteasome-mediated destruction. These small proteins can be expressed in bacteria and purified in high yield to provide a reagent that, as a single component, can be introduced into cells to induce degradation of the target protein within minutes (Figure 6) and with minimal off-target degradation (Figure 5). We envisage two distinct modalities for the use of ARMeD. In the first approach, the RING domain could be fused to one of the many nanobodies available to mediate destruction of the target protein. A recent analysis indicated that almost 800 single domain antibodies, or nanobodies, have been characterized and the sequences made available to the scientific community (Wilton et al., 2018). This number is increasing rapidly as these nanobodies are being used in applications including structural biology (Pardon et al., 2014), super-resolution microscopy (Pleiner et al., 2015), and intracellular signaling studies (Prole and Taylor, 2019). As coverage of the proteome increases and the nanobody database expands, it should be possible to search the database for a nanobody to the protein to be targeted, have a G-block synthesized corresponding to the sequence of the nanobody, and have the nanobody-RING fusion expressed in bacteria and purified ready for knockdown studies in a matter of days. The advantage of this approach is that protein depletion can be achieved without any prior manipulation of the cells under study. However, when nanobodies of the target protein are not available, an alternative approach is to either use a pre-existing cell line containing a GFP tagged protein or to generate an endogenously GFP-tagged protein using CRISPR/cas9 technology. The GNb-2xRING nanobody-RING fusion could then be used as a single reagent to induce the degradation of any GFP-tagged protein. This could be done in almost any eukaryotic organism as the RING domain of RNF4 is highly conserved and human RNF4 was shown to function in yeast (Sun et al., 2007). As the ARMeD system appears to display minimal off-target destruction, target selection is dependent on the unique specificity of the nanobody. This represents a major advantage of the nanobody-based approach as the system is capable of selective degradation of post-translationally modified proteins (Chirichella et al., 2017) or the mutant proteins (oncogenes) responsible for cancer (Quevedo et al., 2018). Although considerable challenges remain to be overcome in the delivery of proteins, the therapeutic application of the ARMeD approach may have use in the destruction of disease-causing proteins.

## STAR★Methods

### Key Resources Table

**Table:** 

REAGENT or RESOURCE	SOURCE	IDENTIFIER
**Antibodies**		

Rabbit monoclonal anti-camelid HRP	GenScript	A01681
Rabbit polyclonal anti-camelid HRP	GenScript	A02016
Mouse monoclonal anti-α-tubulin	ThermoFisher	PA5-22060
Mouse monoclonal anti-GFP	Roche	1814460001
Mouse monoclonal anti β-actin	Sigma-Aldrich	A5316
Sheep polyclonal anti NEDP1	Bailly et al., 2019	N/A
Rabbit monoclonal anti NEDD8	Epitomics	1571-1
Rabbit monoclonal anti TRIM28	Cell Signaling Technology	4124

**Chemicals, Peptides, and Recombinant Proteins**		

Doxycycline Hydrochloride	Sigma-Aldrich	D3447
Proteasome inhibitor MG132	Sigma-Aldrich	C2211
Proteasome inhibitor Bortezomib	Selleckckem	PS0341
Autophagy inhibitor Bafilomycin A1	ENZO	BML-CM110-0100
the KOD Hot Start DNA Polymerase	Merck Millipore	71086
FastDigest Restriction enzymes	Thermo Fisher/Fermentas	N/A
Rapid DNA Ligation Kit	Roche	11635379001
DAPI Stain	Thermofisher	62248
HCS cellmask red stain	Thermofisher	H32712
6His-GFP-Sumo1	This study	N/A
6His-NEDP1	This study	N/A
RNF4 2xRING	This study	N/A
Ubiquitin	This study	N/A
UbcH5a	This study	N/A
MBP-NEDD8-Ub	This study	N/A
GNb-1xRING	This study	N/A
GNb-2xRING	This study	N/A
GNb-1xmtRING	This study	N/A
GNb-2xmtRING	This study	N/A
NNb2-1xRING	This study	N/A
NNb2-2xRING	This study	N/A
NNb7-1xRING	This study	N/A
NNb7-2xRING	This study	N/A
NNb9-1xRING	This study	N/A
NNb9-2xRING	This study	N/A

**Critical Commercial Assays**		

Lipofectamine 3000	Life Technologies	L3000015
Lipofectamine RNAiMAX	Life Technologies	13778150
Neon Transfection System 100 μL Kit	Life Technologies	MPK10025/MPK10096
Neon Transfection System 10 μL Kit	Life Technologies	MPK1096/ MPK1025
mMESSAGE mMACHINE T7 ULTRA Transcription Kit	Thermo Fisher	AM1345
MEGAclear Transcription Clean-Up Kit	Thermo Fisher	AM1908
QIAquick MinElute Gel Extraction Kit	QIAGEN	28604
Pierce BCA Protein Assay Kit	Thermo Fisher	23225
E.Z.N.A Total RNA Kit	VWR	R6834
First Strand cDNA Synthesis Kit	Thermo Fisher	K1612
PerfeCTa® SYBR® Green RT-PCR kit	Quanta Biosciences	95054-500

**Deposited Data**		

Study original experimental raw data deposited to Mendeley.	https://dx.doi.org/	https://doi.org/10.17632/ygvn5hmv78.1
Mass spectrometry data deposited to the ProteomeXchange Consortium	https://www.ebi.ac.uk/pride	PXD018113

**Experimental Models: Cell Lines**		

HeLa	ATCC	CCL-2
HEK293	ATCC	CRL-1573
HeLa Flp-in/T Rex	Thermo Fisher	R71407
HeLa Flp-in/T Rex GNb-1xRING	This study	N/A
HeLa Flp-in/T Rex GNb-2xRING	This study	N/A
HeLa Flp-in/T Rex GNb-1xRING EYFP-PARG	This study	N/A
HeLa Flp-in/T Rex GNb-2xRING EYFP-PMLIII	This study	N/A
HeLa Flp-in/T Rex NNb2-1xRING	This study	N/A
HeLa Flp-in/T Rex NNb2-1xmtRING	This study	N/A
HeLa Flp-in/T Rex NNb9-2xRING	This study	N/A
HeLa Flp-in/T Rex NNb9-2xmtRING	This study	N/A
HEK293 EYFP-SP100	This study	N/A
MCF7 GFP-tubulin	Gift from Prof. Jason Swedlow	N/A

**Oligonucleotides**		

DNA primers: see Table S3		N/A

**Recombinant DNA**		

pOG44	Invitrogen	V600520
pCDNA5 FRT TO	Invitrogen	V652020
pCDNA5 FRT TO GNb-1xRING	This study	N/A
pCDNA5 FRT TO GNb-2xRING	This study	N/A
pCDNA5 FRT TO NNb2-1xRING	This study	N/A
pCDNA5 FRT TO NNb2-1xmtRING	This study	N/A
pCDNA5 FRT TO NNb9-2xRING	This study	N/A
pCDNA5 FRT TO NNb9-2xmtRING	This study	N/A
pCDNA5 FRT TO NNb7-1xRING	This study	N/A
pCDNA5 FRT TO NNb7-2xRING	This study	N/A
pLou3 GNb-1xRING	This study	N/A
pLou3 GNb-1xmtRING	This study	N/A
pLou3 GNb-2xRING	This study	N/A
pLou3 GNb-2xmtRING	This study	N/A
pLou3 NNb2-1xRING	This study	N/A
pLou3 NNb2-2xRING	This study	N/A
pLou3 NNb7-1xRING	This study	N/A
pLou3 NNb7-2xRING	This study	N/A
pLou3 NNb9-1xRING	This study	N/A
pLou3 NNb9-2xRING	This study	N/A
pEFIRES-P-EYFP-C1	This study	N/A
pEFIRES-P-EYFP-C1 PARG	This study	N/A
pEFIRES-P-EYFP-C1 RNF146	Medical Research Council Protein Phosphorylation and Ubquitilation Unit Reagents and services https://mrcppureagents.dundee.ac.uk/reagents-cdna-clones/overview	DU20596
pEFIRES-P-EYFP-C1 PEX10	Medical Research Council Protein Phosphorylation and Ubquitilation Unit Reagents and services https://mrcppureagents.dundee.ac.uk/reagents-cdna-clones/overview	DU20598
pCMV EYFP-IRESpuro PMLIII	This study	N/A

**Software and Algorithms**		

IN Cell Developer Toolbox	GE Healthcare	version 1.91 build 2206
MaxQuant software	Cox and Mann, 2008	version 1.6.1.0
Skyline Targeted Mass Spec Environment	https://skyline.ms/project/home/software/Skyline/begin.view	Version 19.1.0.193
ImageJ	NIH, https://imagej.nih.gov/ij/	Version 1.49K

### Resource Availability

#### Lead Contact

Further information and requests for resources and reagents should be directed to and will be fulfilled by the Lead Contact, Ronald T. Hay (r.t.hay@dundee.ac.uk).

#### Materials Availability

All unique/stable reagents generated in this study are available from the Lead Contact with a completed Materials Transfer Agreement.

#### Data and Code Availability

The original experimental raw data of this study have been deposited to Mendeley (https://dx.doi.org) with the identifier https://doi.org/10.17632/ygvn5hmv78.1.

The mass spectrometry proteomics data have been deposited to the ProteomeXchange Consortium via the PRIDE [1] partner repository with the dataset identifier PXD018113

The following ProteomeXchange ID has been reserved for our proteomic data: PXD016193 (http://proteomecentral.proteomexchange.org/cgi/GetDataset?ID=PXD016193). The access URL (http://panoramaweb.org/project/Panorama%20Public/2019/U%20of%20Dundee%20Hay%20Lab%20-%20NEDP1_ARMeD_knockdown/begin.view?) is the unique identifier of our data on Panorama Public.

### Experimental Model and Subject Details

HeLa and HEK293, (ATCC) were cultured in DMEM-Glutamax medium(Life Technologies 61965) supplemented with 10% Calf Serum and penicillin-streptomycin. HeLa, Flp-in/T.rex cells (Life Technologies) were cultured in Minimum essential Medium – Eagle EBSS, with L-Glutamine (Lonza 12-611F) supplemented with 10% Calf Serum and penicillin-streptomycin. HeLa Flp-in/T Rex (Life Technologies) grown in mono layer were transfected with each of the GFP or NEDP1 nanobody- wild-type or mutant RING/RING-RING fusion constructs descried above, along with the Flp recombinase vector pOG44, using Lipofectamine 3000 (Life Technologies) according to the manufacturers’ instructions and selected with hygromycin at 100 μg/ml. Thereafter, stable cell populations were maintained in growth medium containing hygromycin (50 μg/ml) and blasticidin (5 μg/ml). Cells stably transfected with pCDNA5 FRT TO-GNb-1xRING or pCDNA5 FRT TO-GNb-2xRING were subsequently transfected with pEFRE-P-EYFP-C1-PARG or pCMV EYFP-IRESpuro PMLIII, respectively, selected with 1 μg/ml puromycin and maintained in growth medium containing puromycin (0.5 μg/ml), hygromycin (50 μg/ml) and blasticidin (5 μg/ml). Following confirmation of the YFP fusion protein degradation in response to doxycycline treatment homogeneous populations were selected by diluting the cell cultures to 1 cell/well and growing them under selection in 96-well plates until the appearance and growth to confluence of single colonies. HEK293 cells stably expressing EYFP-SP100 were kindly provided by Ellis Jaffray. MCF7 cells stably expressing a GFP-tubulin fusion were a kind gift from Jason Swedlow. For doxycycline induction experiments cells were treated with 1 μg/ml doxycycline (Sigma). For experiments involving proteasome and/or autophay inhibition, 10 mM MG132 (Sigma; C2211), 1 μM Bortezomib (Selleckckem PS0341) or 100 nM Bafilomycin A1 (ENZO BML-CM110-0100), or a corresponding volume of DMSO was added to the medium 90 minutes prior to starting the experiment. All cell lines used are of human origin. HeLa and MCF7 are female. The HEK293 line is most likely female due to the presence of multiple X chromosomes and no detectable Y chromosome (Lee and Mendell, 2020). Cells used were negative when tested for mycoplasma.

### Method Details

#### Plasmids

The coding sequences of a camelid-derived single-domain antibody (nanobody) (PDB accession 3K1K_C) raised against the green fluorescence protein (GFP), was generated synthetically (GeneArt, Thermofisher) with a 5′ HindIII and 3′ NheI restriction recognition sites. The coding sequence for residues 75-194, including the RING domain (residues 131-194) of Rattus norvegicus RNF4 (accession: NM_019182, UniProtKB - O88846) were amplified from previously generated expression constructs in pLou3 (Plechanovová et al., 2011) by the polymerase chain reaction (PCR) with 5′ NheI and 3′ BamHI-NotI restriction sites. The synthetically generated GFP nanobody and the RNF4 75-194 were ligated into the pCDNA5 FRT TO vector (Life Technologies) via a 3 point ligation HindIII-NheI-NotI, resulting in a GFP nanobody-wild-type RNF4 RING fusion (GNb-1xRING). To create a linear fusion of GNb-1xRING and the RING domain of RNF4 the RING domain was PCR-amplified with a 5′ BamHI sand a 3′ NotI restriction sites and inserted between the respective sites in GNb-1xRING and the resulting fusion was denoted “GNb-2xRING.” To generate nanobody-RING fusions targeting the NEDD8 specific protease NEDP1 (SENP8; accession NM_145204; UniProtKB - Q96LD8) the coding sequences for three nanobodies raised against this protein, NEDP1 nanobody 2,7 and 9, were produced by gene synthesis (GeneArt, Thermofisher) with 5′ HindIII and 3′ NheI restriction sites and sub-cloned into the pCDNA5 FRT TO-GNb-1xRING and pCDNA5 FRT TO-GNb-2xRING described above, replacing the GFP nanobody sequence and resulting in the pCDNA5 FRT TO-nanobody-1x and 2xRING listed in the Key Resources Table. Subsequently, the coding sequences for RNF4 RING and RNF4 RING-RING containing M140A and R181A mutations within the RING domain sequences were PCR-amplified, starting from residue 131 as above, from previously generated constructs (Plechanovová et al., 2011) with 5′ NheI and 3′ NotI restriction sites and sub-cloned into the NEDP1 nanobody-RING constructs to replace the wild-type RNF4 RING sequences, resulting in in pCDNA5 FRT TO-NNb2, 7, 9 1xmtRING and 2xmtRING. All nanobody-RING fusions contained an alanine-serine linker between the nanobody and the RNF4 sequence, and all nanoody-RING-RING fusion constructs contained a single glycine linker between the two RINGs. Bacterial expression constructs from all nanobody-RING and RING-RING fusions were created by PCR amplification of the fusion sequences from the above constructs with 5′ NcoI and 3′ XhoI sites and sub-cloned between the NcoI and SalI sites of pLou3 with N-terminal 6His-MBP tag and TEV protease cleavage site. To create a mammalian overexpression cDNA construct for Poly ADP ribose glycohydrolase (PARG; NM_003631; UniProtKB - Q86W56) with N-terminal enhanced yellow fluorescence protein (EYFP) tag we first created pEFIRES-P-EYFP-C1 by inserting the EYFP sequence after PCR-amplification from pEYFP-C1 (Invitrogen) with the upstream NheI site and adding in-frame 3′ SpeI and XhoI sites, into the Nhe I and XhoI I sites of the plasmid vector pEFIRES-P (Hobbs et al., 1998). We then PCR-amplified the PARG coding sequence was from cDNA clone MGC:57711, IMAGE:6064831 with 5′ SpeI and 3′ NotI restriction sites and cloned it into the respective sites of pEFIRES-P-eYFP-C1. RNF146 (NM_030963.2) and PEX10 (NM_002617.3) cDNA clones in pEFIRES-P-eYFP-C1 were obtained from the Medical Research Council Protein Phosphorylation and Ubiquitylation Unit Reagents and services (https://mrcppureagents.dundee.ac.uk/reagents-cdna-clones/overview). pCMV eYFP-IRESpuro PMLIII was kindly provided by Ellis Jaffray. The sequences of all the oligonucleotide primers used for DNA cloning are shown in Table S3. All constructs were verified by DNA sequencing (https://www.dnaseq.co.uk).

#### siRNA transfections

Cells were transfected with a pool containing an equimolar amount of four siRNA duplexes targeting NEDP1 (SENP8, accession: NM_145204, Dharmacon ON-TARGETplus; SENP8, 1- GAUCACGUCAGUUUCAUCA; SENP8, 2- UGAGUUACAUGGACAGUCU; SENP8, 3- CCAACAGUCAGUUUCAUGA; SENP8, 4- GGGAUGUACGUGAUAUGUA) to a final concentration of 10 nM, or a non-targeting control duplex (siNT) at the same concentration using Lipofectamine RNAiMAX (Life Tecnologies) according to the manufacturer’s instructions. Total protein extracts were prepared 72 hours following transfection.

#### Cell lysis and immunoblot analysis

Cells were washed in PBS and whole-cell extracts were prepared by lysis in 2x Laemmli sample buffer (5% w/v SDS, 150 mM TRIS-HCl pH 6.7, 3% v/v glycerol, 0.01% w/v bromophenol blue) and heated at 95°C for 5 mins. Protein concentration was measured using the Pierce BCA Protein Assay Kit (Thermo Fisher 23225) according to the manufacturers’ instructions. Then b-mercaptoethanol was added to 700 mM and the cell lysates were separated in NuPAGE 4%–12% Bis-Tris gels (Thermo Fisher) and transferred to PVDF membrane. Primary antibody incubations were performed in PBS with 2% BSA and 0.1% Tween-20. For the secondary antibody incubations the 5% milk was used instead of the BSA. Primary antibodies used were mouse anti-GFP (Roche 11814460001, 1:1000), sheep anti-NEDP1 (Bailly et al., 2019, 1:1000), rabbit anti-NEDD8 (Epitomics 1571-1, 1:1000), sheep anti-RNF4 (homemade, 1:1000) and anti-alpha tubulin (ThermoFisher PA5-22060, 1:10000). HRP-coupled secondary anti-mouse, anti-rabbit and anti-sheep were purchased from Sigma. HRP-rabbit anti-camelid VHH was purchased from GenScript (A01681). The signal was detected by Pierce enhanced chemiluminescence (ThermoFisher 32106) and X-ray films.

#### RNA Isolation and quantitative RT-PCR

Total RNA was isolated using the E.Z.N.A Total RNA Kit (VWR R6834) with in-column DNase digestion following the manufacturer’s protocol. cDNA was prepared using the First Strand cDNA Synthesis Kit (ThermoFisher K1612) and quantitative RT-PCR was performed using PerfeCTa® SYBR® Green (Quanta Bioscience) according to the supplier’s protocol. qPCR was performed in either a 96 or 384-well format using Biorad CFX96/CFX384 or Applied Biosystems QuantstudioFlex 6 thermal cycler’ Thermal cycling conditions were an initial denaturation step of 95°C for 10 mins, and then 44 cycles of 95°C for 15 s, 60°C for 60 s followed by 95°C for 10 s and a melt curve of 65°C to 95°C. The primers were designed to produce amplicons crossing the nanobody-RNF4 boundary. Standard curves were produced for each amplicon-specific primer set and for the control gene Beta-2-Microglobulin (B2M) primers. RNA was always prepared from three independent cultures (replicates) representing each experimental condition and the PCR reaction was performed in duplicate for each RNA sample. The data were analyzed by the software accompanying the used instrument and presented after normalization against the control gene.

#### *In vitro* transcription of ARMeD mRNAs

*In vitro* transcription, capping and polyadenylation of ARMeD mRNAs was performed using the mMESSAGE mMACHINE T7 ULTRA Transcription Kit (Thermo Fisher AM1345) and purified with the MEGAclear Transcription Clean-Up Kit (Thermo Fisher AM1908) according to the manufacturers’ instructions. DNA templates were PCR amplified from the corresponding plasmids using the KOD Hot Start DNA Polymerase (Merck Millipore 71086) and purified by agarose gel electrophoresis using the QIAquick MinElute Gel Extraction Kit (QIAGEN 28604). The used primer sequences are shown in Table S3.

#### High-content imaging

Cells were seeded in black, clear-bottomed 96-well plates (Greiner μClear) in 100 μl culture medium for 24 hours prior to the experiment.at the end of the experiment cells were washed twice with PBS, fixed with 4% formaldehyde and stained with 0.2 μg/ml DAPI (Thermofisher 62248) in combination HCS cellmask red stain (Thermofisher H32712). 100 μl of PBS was dispensed into wells and plates were sealed with an adhesive aluminum foil seal. Imaging was performed using an IN Cell 2000 microscope (GE Healthcare) to acquire three fields of view per well with a 10 or 20 × lens (Nikon), capturing DAPI, CellMask and EYFP. All images displayed in this paper for comparing the cellular response to different biological treatments were adjusted by applying identical visual parameters using ImageJ (NIH). Image analysis was performed by IN Cell Developer Toolbox version 1.91 build 2206 (GE Healthcare), using protocols designed to identify EYFP-PARG or EYFP-PML/EYFP-SP100 inclusions by region-growing or multi-scale top hat transformation, respectively. To measure EYFP, nuclear intensity was used as the most robust parameter, while the measure of total organelle area per cell nucleus was selected as the most discriminatory for changes in EYFP-PML and EYFP-SP100 following treatment. For transfection efficiency calculations the cell/background intensity measure was found to give the most robust results and a threshold of 1.075 was used as the lower limit to be achieved by transfected cells. Data were obtained for > 20000 cells per condition and the presented data represent the mean ± SD. For degradation kinetics the time required to degrade 50% of the initial protein amount (t½) was deduced from the exponential equation resulting from plotting the obtained intensity or total area values against time.

#### Protein expression and purification

Nanobody fusion proteins were expressed in *E. coli* SHuffle cells (New England BioLabs) at 20°C overnight after induction with 0.1mM IPTG. His6-MBP tagged fusion proteins were purified by Ni-NTA (QIAGEN) affinity chromatography and dialyzed overnight in 50mM Tris HCl pH7.5, 150 mM NaCl, 0.5mM TCEP buffer. To remove the His6-MBP tag, fusion proteins were incubated with TEV protease, followed by Ni-NTA affinity chromatography to remove any uncleaved His6-MBP tagged proteins, free His6-MBP tag and TEV protease (also His6-tagged). Purified untagged Nanobody fusion proteins were then dialyzed against 50 mM Tris HCl pH 7.5, 150 mM NaCl 0.5 mM TCEP further purified by gel filtration (Superdex75) and flash-frozen in liquid nitrogen prior to storage at −80°C.

#### Pull-down assay

The interaction between GFP nanobody-RING fusion proteins and GFP was studied using a pull-down experiment. His6-EGFP-SUMO1(20μM) was incubated for ∼30 min at room temperature with RNF4 RING-RING fusion (negative control), Nanobody-RING, Nanobody-RING-RING or Mutants (20μM) in a total volume of 200 ul containing 50 mM Tris.Cl pH7.5, 150 mM NaCl, 0.5 mM TCEP. 50ul of Nickel beads were added in mixture and continue to incubate for 30 minutes. Nickel beads were collected on the bottom of the tube by centrifugation and samples were taken from the supernatant. Beads were washed 3 times with 0.5 mL of binding buffer. Bound proteins were eluted from the beads by addition of SDS-PAGE loading buffer and analyzed by SDS-PAGE.

In the pull-down experiments shown in Figure 7A, His6-NEDP1(20μM)was incubated for 5 min at room temperature with RING-RING (negative control), or NEDP1 nanobody2-2xRING or Nanobody2-RING (∼20μM) immobilized on Nickel beads (50ul) in a total volume of 200ul. Subsequently, beads were washed once as described above and bound material was eluted with SDS-PAGE loading buffer, analyzed by SDS-PAGE.

#### *In vitro* NEDP1 inhibition assay

NEDP1 (50 nM) was preincubated with nanobody fusion proteins (60nM) at 25 oC for 15 min in reaction buffer (50 mM Tris-HCl pH 7.5, 150 mM NaCl, 0.2mM TCEP,). Then substrate MBP-NEDD8-Ub was added to a final concentration of 4uM and incubated for 1hour at 37 oC. The reaction was terminated by adding loading buffer and boiling for 5 min, followed bySDS-PAGE and Coomassie staining for visualization.

#### Lysine discharge assay

UbcH5a∼Ub linked conjugate was prepared by mixing the following components for 20 min at 37°C: 120 μM UbcH5a, 100 μM Ub, 0.2 μM Ube1, 50 mM Tris pH 7.5, 150 mM NaCl, 5 mM ATP, 5 mM MgCl2, 0.5 mM TCEP, 0.1% NP40. Apyrase (4.5 U ml−1, New England BioLabs) was then added to the reaction to deplete the ATP. The thioester was then mixed in a 1:1 ratio with test proteins, 10mM L-lysine buffered with 50 mM Tris pH 7.5, 150 mM NaCl, 0.1% NP40, 0.5 mM TCEP. The final concentration of each component is about 30 μM thioester, 5 mM L-lysine, 50 nM fusion proteins. The reaction was incubated at room temperature, Samples were taken from the reaction mixture at the desired time points, mixed with non-reducing SDS-PAGE loading buffer and analyzed by SDS-PAGE.

#### Microinjection

HeLa Flp-in/T.Rex cells stably expressing YFP-PML were seeded on to glass bottomed dishes (FluoroDish, WPI) and allowed to settle overnight. The cells were then microinjected with 30 μM GNb-2xRING mixed with an equal amount of mCherry (to localize the injected cells) in injection buffer (100 mM glutamic acid, pH 7.2 with citric acid (Izant et al., 1983), 140 mM KOH, 1 mM MgSO4 and 1 mM DTT) as described previously (Prescott et al., 1992). The cells were immediately transferred to the stage of a Zeiss LSM 710 confocal microscope with a 37°C heated stage/chamber and 5% CO2 atmosphere and imaged by time-lapse. A z stack of 7 images (6.3 μm depth) was taken at each time point and one stack was collected every 2 minutes. For each time point the z stacks were compressed into a single maximum intensity projection and the time-lapse data was transferred into Imaris for quantitation. Injected cells were identified using the Texas Red channel and the Mean summed Green, GFP intensity of the injected cells was obtained following subtraction of 5000 units background, based on the uninjected surrounding cells, and plotted for each time point ± sd.

#### Electroporation of cells with ARMeD fusions

Eectroporation was performed using the Neon Transfection System (Thermo Fisher). Cells were washed with PBS and resuspended in Buffer R (Thermo Fisher) at a concentration of 8x10^7^ cells/ml. For HEK293 cells we used 8x10^5^ (10 μl) or 8x10^6^ (100 μl) cells for selection by high content imaging or immunoblotting, respectively. Cells were mixed with 0.03 or 0.12 μg/μl, giving a final concentration of 0.375 pg or 1.5 pg of the recombinant fusion protein/cell, or PBS and electroporation was performed in 10 or 100 μl electroporation tips according to the manufacturers’ instructions with 2 pulses at 1400V for 20 ms each. Similar conditions were applied for MCF7 except that 5x10^6^ cells were used in the 100 μl tips and the electroporation was performed with 2 pulses at 1100V for 30 ms. Immediately after electroporation the cells were transferred to growth medium with10% FBS but without antibiotics. For immunoblot analysis aliquots were taken at the desired time points, and the reaction stopped by cooling on ice and centrifugation at 90xg for 10 min at 4°C followed by cell lysis in 2x Laemmli sample buffer. For high content imaging aliquots were taken at the desired time point and the degradation reaction stopped immediately by adding the cells to an equal volume 8% formaldehyde in a black, clear-bottomed 96-well plates (Greiner μClear) followed by centrifugation at 90xg for 20 min at room temperature, washing, and DAPI staining as described. To determine transfection efficiency, cells were co-electroporated with a mCherry labeled protein and high content data were collected and analyzed as described above.

#### Quantitative proteome analysis

To monitor changes to the cellular proteome during induction of the NNb2-1xRING construct, a quantitative proteomics experiment was performed. Two cultures of HeLa Flp-in/T-REX NNb2-1xRING cells were grown in either ‘Light’ or ‘Heavy’ SILAC medium as described (Ong and Mann, 2007). Briefly, cells were grown in Dulbecco’s modified Eagle’s medium lacking all amino acids except L-lysine and L-arginine, which were supplemented with either isotopically typical lysine or arginine (‘Light’), or ^13^C_6_, ^15^N_2_-lysine and ^13^C_6_,^15^N_4_-arginine (‘Heavy’). After full label incorporation, two 100mm dishes of each labeled form of the cells were used for the SILAC comparisons shown in Figure 4A. By this design, two parallel comparisons differing only by the SILAC labels could be used to monitor the effect of Dox treatment on the cellular proteome. After treatment with Dox or not, cells were washed twice with PBS and individual whole cell extracts were made by addition of 1.2x LDS sample buffer containing reducing agent (Invitrogen) followed by sonication and heating to 70°C for 5 minutes. Protein concentrations were calculated by Bradford’s method and 40 μg total protein was prepared for each SILAC mixture by mixing 1:1 (w:w) the appropriate extracts. These two mixes were fractionated by 4%–12% acrylamide SDS-PAGE (Invitrogen NuPAGE Bis-Tris gels – MOPS buffer), and each lane of the Coomassie-stained gel excised into 16 equally sized slices (Figure 4B). Gel pieces were subjected to in gel tryptic digestion (Shevchenko et al., 2006), employing both reduction with DTT and alkylation with chloroacetamide prior to digestion. Extracted peptides were dried down under vacuum and resuspended in 35μL 0.1% TFA 0.5% acetic acid.

Peptide samples were analyzed twice. First, 18μL of each peptide sample was analyzed by LC-MS/MS on a Q Exactive mass spectrometer (Thermo Scientific) coupled to an EASY-nLC 1000 liquid chromatography system (Thermo Scientific) via an EASY-Spray ion source (Thermo Scientific). Peptides were fractionated on a 75 μm x 500 mm EASY-Spray column (Thermo Scientific) over a 240 minute gradient. For all runs precursor ion full scan spectra were acquired over (m/z 300 to 1,800) with a resolution of 70,000 at m/z 400 (target value of 1,000,000 ions, maximum injection time 20 ms). Up to fifteen data dependent MS2 spectra were acquired with a resolution of 35,000 at m/z 400 (target value of 500,000 ions, maximum injection time 120 ms). Ions with unassigned charge state, and singly or highly (> 8) charged ions were rejected. Intensity threshold was set to 2.1 × 10^4^ units. Peptide match was set to preferred, and dynamic exclusion option was enabled (exclusion duration 15 s). The second MS runs used 90minute fractionation gradients with a top 10 method, 40 s dynamic exclusion period and loaded 16 μL peptide solution per slice. All other parameters were the same as the first run. For both runs samples of recombinant NEDP1 tryptic peptides were also analyzed at the end of each run batch. This was in an attempt to identify peptides in the SILAC samples using spectral matching in MaxQuant, although this was unsuccessful.

The 66 raw MS data files were processed using MaxQuant software (version 1.6.1.0) (Cox and Mann, 2008), and searched against UniProtKB human proteome (canonical and isoform sequences; downloaded in April 2019), plus a fasta file containing the sequence of the induced NEDP1 nanobody-RING construct:

Most settings were left at default but briefly: The appropriate SILAC labels were selected and enzyme specificity was set to Trypsin (three missed cleavages). Importantly the re-quantify option was selected, without which peptides with missing SILAC counterpart peptides are not quantified and so proteins with large changes among conditions are not reported. This was necessary to obtain ratios for the nanobody construct itself. Carbamidomethylation of cysteines was set as a fixed modification and oxidation of methionine and acetylation of protein N-termini were set as variable modifications. Second peptide data was requested. The ‘match between runs’ option was selected to maximize the numbers of common identifications between the two SILAC mixes in identical or adjacent gel bands. Minimum peptide length was set to seven amino acids and a maximum peptide mass was 4600 Da. A false discovery rate of 1% was set as a threshold at both protein and peptide level, and a mass deviation of 6 parts per million was set for main search and 0.5 Da for MS2 peaks. For the first MS run files slices were numbered 1 to 16 in the “Fraction” column of the experimental design template file, and from 101-116 for the second run, so spectral matching did not occur between MS runs. All slices from the same SILAC mix were given the same ‘Experiment’ name to separate the ratio data into the two mixes (A & B) and aggregate data from both MS runs.

The proteinGroups.txt file was filtered for entries from the decoy database, those identified by modified peptide(s) only, potential contaminants, and those with SILAC ratio variability > 140% in either MixA or MixB. This left 5635 proteins, of which 4907 had SILAC ratios reported in both mixes. Outliers in each mix were defined using SigB in Perseus, (v 1.6.1.1) (Tyanova et al., 2016) using the ‘both sides’ method, truncated using a Benjamini-Hochberg FDR threshold of 5%. The 7 proteins ultimately defined as having significantly affected protein abundance by Dox treatment (Figures 5C and 5D) were that that met the SigB cutoff in both SILAC mixes and whose increase or decrease in response to Dox consistently in both. This left four proteins. Summary of these data can be found in Table S1, worksheet “Summary_by_mix.”

The slice-by-slice comparison to monitor protein changes throughout the gel used just the 240 minute MS run data. In MaxQuant, every raw file was given a unique ‘Experiment’ name so protein data was separated by slice in the final output files. In this instance ‘requantify’ was turned off to ensure detection in any slice was not made by matching across files. All other MaxQuant setting were left as default. Summary of these data can be found in Table S1, worksheet “Summary_by_slice.”

Four peptides derived from NEDD8 itself were assigned by MaxQuant to the fusion protein NEDD8-MDP1 [UniProtKB - E9PL57 (E9PL57_HUMAN)]. One NEDD8 peptide not shared with this construct was assigned to NEDD8 [UniProtKB - Q15843 (NEDD8_HUMAN)]. For the slice-by-slice analysis, to extract protein level data for NEDD8 only, the five individual NEDD8 peptides intensity data were summed and these values entered into the proteinGroups table under the protein name “NEDD8 (MHT curated).” This included data for the peptides (TLTGKEIEIDIEPTDKVER, EIEIDIEPTDKVER, IKERVEEKEGIPPQQQR, VEEKEGIPPQQQR, and ILGGSVLHLVLALR). The original entries for NEDD8-MDP1 and NEDD8 were deleted. In the non slice-by-slice (total protein change) analysis, the original entries were left as reported by MaxQuant due to there being no evidence of abundance change upon Dox treatment. Notably, peptides derived from the MDP1 portion of the NEDD8-MDP1 fusion were found exclusively in slice 14 in both mixes (Table S1 “NEDD8-MDP1 peptides” worksheet). As MDP1 itself has length 176 amino-acids and expected mass 20.1 kDa and slice 14 encompassed the 19-24kDa region of the gel (Figure 4B), this confirms NEDD8 and MDP1 peptides were falsely assigned to the NEDD8-MDP1 fusion protein rather than the individual proteins.

#### Protein copy number per cell calculation

The protein copy number per cell in the proteomic analysis was estimated using the “Proteomic Ruler” plugin (Wiśniewski et al., 2014) in Perseus v1.5.1.6. Calculations were based on the summed H and L intensities in each SILAC mix separately to give two copy number per cell values for each protein. The final reported value was the average of the two. The same human proteome fasta file was used for calculations as was used for MaxQuant processing. The protein annotation step used median of all IDs for sequence length, average molecular mass and theoretical trypsin peptides. Copy number estimates considered the two mixes separately, used the average molecular mass and was corrected by the number of theoretical peptides calculated at the previous step. Ploidy was set to 3.4 (HeLa cells) and a total cellular protein concentration of 100 g/l. These values resulted in total protein calculations of 187 and 193 pg/cell and cell volumes of 1869 and 1932 fl, which is broadly in agreement with expectation according to the bionumbers database (https://bionumbers.hms.harvard.edu).

#### Targeted proteomic analysis of NEDP1

To monitor changes to NEDP1 levels in cells a Parallel Reaction Monitoring (PRM) (Peterson et al., 2012) method was employed. Tryptic peptide samples were prepared from parental HeLa and cells expressing the ARMeD construct for NEDP1 or GFP ± doxycycline 1ug/ml (See Table S2, worksheet “PRM Experimental Design”), as well as from recombinant NEDP1. To define a NEDP1 peptide inclusion list (Table S2, worksheet “Inclusion list”) tryptic peptides derived from 500ng of digested recombinant NEDP1 protein were analyzed first in a data-dependent analysis (DDA) by LC-MS/MS on the Q Exactive setup described above. Next, to define a list of cellular peptides to control for sample loading in the PRM analysis, a mixed sample was generated by pooling tryptic peptides from PARENTAL, NEDP1, and GFP control cell lines ± doxycycline, and 1ug was run in triplicate immediately following the recombinant samples. Discovery experimental design is described in Table S2, worksheet “Discovery experimental design.” iRT peptides were spiked into all samples (Biognosys Cat# Ki-3002-2), and both the iRT and control peptides were added to the inclusion list. MS runs were acquired over identical 90 minute gradients (Table S2, worksheet “QE methods”) with flow rate 20 ul/min, buffer A HPLC-grade water 0.1% formic acid, and buffer B mass spectrometry-grade acetonitrile 0.1% formic acid. DDA methods consisted of precursor ion full scan acquired over m/z range of from 300 to 1,800 with a resolution of 70,000 at m/z 200, a target value of 1,000,000 ions, and maximum injection times of 20 ms. Up to 4 data dependent MS2 spectra were acquired with a resolution of 70,000 at m/z 200, a target value of 1,000,000 ions, and a maximum injection time 300 ms. Ions with unassigned charge state, and singly or highly (> 8) charged ions were rejected. Intensity threshold was set to 2.0 × 10ˆ4 units. Peptide match was set to preferred, and dynamic exclusion to 40 s. The run was conducted in positive ion mode.

DDA raw files were searched with MaxQuant v1.6.1.0 (Cox and Mann, 2008) for NEDP1 nanobody-RING, iRT peptides, and the UniProtKB human proteome (canonical and isoform sequences; downloaded in April 2013) using 1% FDR for both proteins and peptides, trypsin digestion with 4 max missed cleavages, minimum peptide length of 5 amino acids, and maximum peptide mass of 10,000 Da. Calculate Peak Properties was selected, a threshold score of 40 was applied, and all other settings left as default. All discovery runs were used to generate the inclusion list, parameter file, and msms.txt data file for PRM. The inclusion list combined 24 NEDP1 peptides and 11 iRT peptides, as well as 63 high scoring human protein peptides for use as sample loading controls.

PRM was performed on 12 ul (approximately one third) of each of the 18 cellular peptide samples described above, each spiked with iRT control peptides, using the same 90 min elution gradients as the DDA runs. PRM methods included precursor full scans acquired over a scan range of 300-1800 m/z with chromatogram peak widths of 30 s, resolution 70,000 at 200 m/z, a target value of 1,000,000, and a maximum injection time of 100 ms. The inclusion list generated from the DDA data was imported. Up to 12 data dependent MS2 spectra were acquired with a resolution of 70,000 at m/z 200, a target value of 200,000 ions, a maximum injection time of 247 ms, NCE 28, and spectrum data type was set to centroid.

MSConvertGUI v3.0.18270-f64d6f0fe was used to convert PRM .raw files to .mzXML/.wiff format for Skyline analysis. Filters was set to Peak Picking and MS levels was set to 1-2, otherwise settings were left at default.

A blank Skyline document was generated with default settings except where noted in below. A redundant library was kept and a set of 11 Biognosys iRT peptides was used (setting Biognosys-11 iRT-C18). The cut-off score was set at 95, corresponding to a FDR of 5%. The MaxQuant msms.txt file generated from analysis of the DDA runs was imported. The reported iRT graph contained 9 points, with slope 1.7140, intercept −58.1619, and R-squared value 0.993. iRT standard values were recalibrated relative to the peptides added, with a time window of 5 minutes. The sequence of the recombinant NEDP1protease, which contained an additional GA at the N terminus as a result of the TEV cleavage, was added to the target list, as were the inclusion list peptides in FASTA format. The inclusion list contained 100 peptides, 9 of which were sequence duplicates of other inclusion list peptides but differed by charge. Digestion enzyme was set to Trypsin [KR | P], with 1 max missed cleavage. Background proteome was the human proteome plus NEDP1 with GA inserted at the N terminus, digested with trypsin with 1 maximum missed cleavage. The minimum peptide length searched for was 5 amino acids and maximum was 25. Variable modifications selected were carbamidomethylation of cysteines, oxidation of methionine, acetylation at the N terminus, and carboxymethylation at the N terminus. Precursor charges was set to 2-5, ion charges was set to 1-5, and ion types was set to y,b,p. Skyline was set to pick 20 product ions, with a minimum of 5. Minimum m/z was set to 300 and maximum to 1800. Under MS1 filtering the isotope peaks included was set to count, and precursor mass analyzer was set to Orbitrap. Resolving power was set to 70,000 at 200 m/z. Under MS/MS filtering, the acquisition method was set to targeted, and the product mass analyzer was set to Orbitrap. Resolving power was set to 70,000 at 200 m/z. Only scans within 5 minutes of MS/MS IDs were used. 18 PRM .wiff files were imported with sample numbering scheme identical to above, empty proteins and peptides were removed and minimum DOTP threshold was set to 0.75 for NEDP1peptitde analysis. Some chromatogram peak boundaries reported by Skyline were empirically observed to be in error and were manually adjusted. In these instances, the original boundary is shown in the individual Skyline sample chromatograms by magenta shading and the adjusted boundary is indicated by dashed lines. Y series ions from NEDP1 peptides and their charges, masses, and retention times (RT) are available in Table S2, worksheet “NEDP1 Peptide Information.” Analysis of the loading normalization sample resulted in 63 high scoring peptides from 60 proteins which were added to the inclusion list. PRM MS1 peak intensities corresponding to 34 of these peptides were averaged to generate correction factors for sample loading errors. Selection of appropriate sample as well as positive control peptides was based on points across peak > 7, mass error < 4 ppm, and idotp > 0.75. Median number of points across peak for all sample and control peptides was 16.

The 3 NEDP1 peptides detected in the MS2 analysis were LAFVEEK, LEAFLGR, and QVAEKLEAFLGR; however, no QVAEKLEAFLGR fragment ions were detected in the NEDP1 ARMeD construct plus doxycycline cells. To calculate fold-depletion of NEDP1 upon doxycycline induction of the nanobody-ring fusion, 7 fragment ions from the LAFVEEK and LEAFLGR peptides were analyzed. The sums of all fragment intensities from each replicate were calculated. For each set of triplicate samples, the median of these sums was determined. We define the fold-depletion as the ratios of these means, which were taken for each of the following pairwise comparisons: PARENTAL+/−, NEDP1-/PARENTAL-, NEDP1+/PARENTAL-, GFP+/PARENTAL-, GFP-/PARENTAL-, and NEDP1+/ NEDP1-. P values were calculated via two-tailed unpaired t tests using Prism software v8.1.2.

After knockdown the NEDP1+Dox chromatogram peak areas were near background which introduced some challenges in their analyses. When chromatogram peaks were partially overlapped by adjacent spurious peaks the Skyline default was to report N/A instead of an area value. Boundaries for the LEAFLGR peaks in samples 1, 2, 12, and 17 were manually adjusted. This had the effect of avoiding the default N/A and instead reporting a value that was inflated by the adjacent spurious peak. The LAFVEEK peptide y4 ion had an adjacent spurious peak with a significantly different mass (> 20 ppm), and the LEAFLGR peptide y6 ion had an adjacent spurious peak of nearly identical mass. Given that these areas are inflated by the presence of adjacent spurious peaks, any fold-reduction value generated for the knockdown by boundary adjustment as per above will be skewed below the true value.

Only 5 points across peak (PAP) were reported for the sample 12 LEAFLGR peptide, and 2 of the peaks were empirically observed to be indistinguishable from background. Despite being below the threshold of 7, the data was included in order to permit the calculation of a baseline magnitude for the knockdown.

LAFVEEK, LEAFLGR, and QVAEKLEAFLGR peptide sequences were blasted against the human proteome (taxid 9606) using NCBI Blast:Protein Sequence to verify uniqueness. All LAFVEEK, LEAFLGR, and QVAEKLEAFLGR 100% query cover/100% sequence identity matches were unique to NEDP1. NEDP1 protein was queried on phosphosite.org and was found to be potentially acetylated at lysine 146. We were able to detect the relevant peptide (LAFVEEK) and do not expect presence of doxycycline to affect acetylation levels. The reduction in abundance of this peptide upon addition of doxycycline matches that of the LEAFLGR and QVAEKLEAFLGR peptides, which are not known to be acetylated. Samples were not run in a blinded fashion.

### Quantification and Statistical Analysis

Assays were conducted at least in triplicate and presented graphically with SD reported. Statistical analyses and P value calculations were performed by two-tailed unpaired t tests.

## Supplemental Information

Document S1. Figures S1–S6 and Table S3

Table S1 Quantitative Proteome Analysis following NEDP1 ARMeD Induction, Related to Figure 5

Table S2 Targeted Proteomic Analysis of NEDP1, Related to Figures 4 and 5

Document S2. Article plus Supplemental Information
