# Supplementary material for: Antibody RING-Mediated Destruction of Endogenous Proteins
Source: Mol Cell. 2020 Jul 2;79(1):155–166.e9. doi: 10.1016/j.molcel.2020.04.032 (PMC7332993; doi:10.1016/j.molcel.2020.04.032)
Supplement: Document S1. Figures S1–S6 and Table S3 [file mmc1.pdf]

Molecular Cell, Volume *79*

Supplemental Information

Antibody RING-Mediated Destruction of Endogenous Proteins

Adel F.M. Ibrahim, Linnan Shen, Michael H. Tatham, David Dickerson, Alan R. Prescott, Naima Abidi, Dimitris P. Xirodimas, and Ronald T. Hay

A

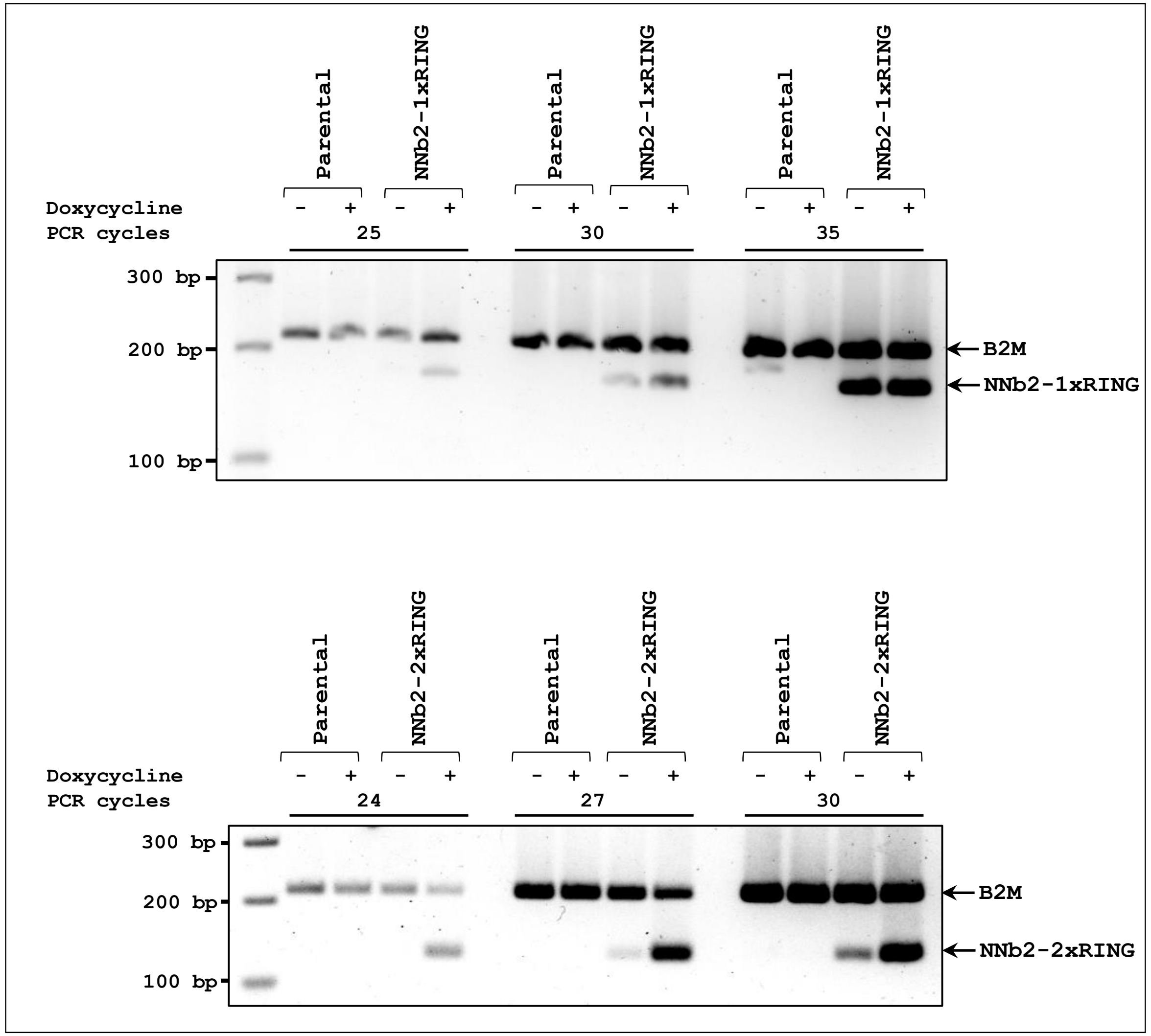

B

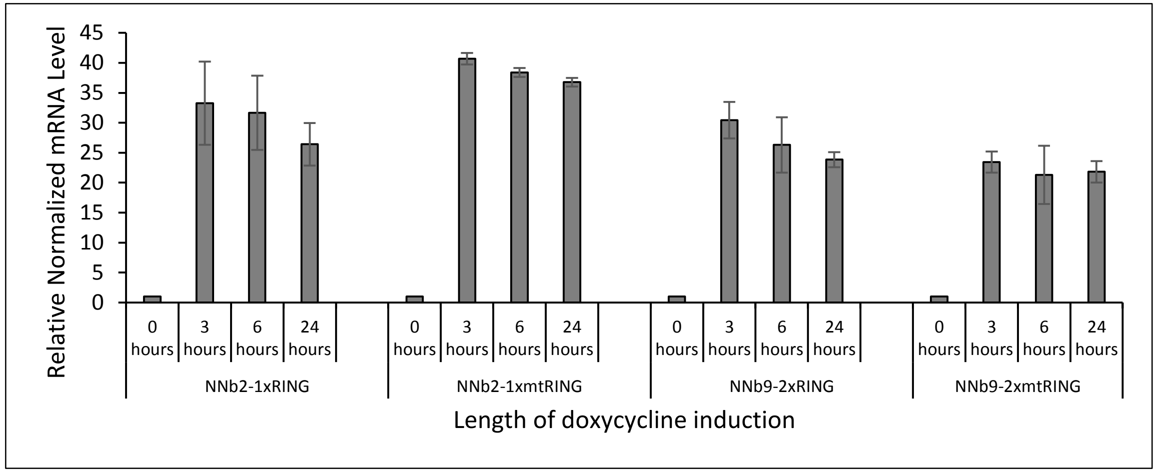

Figure S1. Semi quantitative and quantitative RT-PCR analysis of NEDP1 nanobody-RING fusions in stably transfected Hela Flp-in/T.Rex cells. Related to Figure 4.

(A) Hela Flp-in/T.Rex cells stably transfected with NEDP1 nanobody2-1xRING (NNb2-1xRING; top panel) or NEDP1 nanobody2-2xRING (NNb2-2xRING; bottom panel) were induced with 1 µg/ml Doxycycline for 24 hours (+) or left as untreated controls (-). mRNA levels were analysed by semi-quantitative RT-PCR with beta-2 microglobulin (B2M) as housekeeping control, and the products after stopping the reaction at the indicated PCR cycles were separated on a 2% agarose gel. (B) Quantitative RT-PCR analysis to assess the expression of nanobody-RING fusions in stably transfected Hela Flp-in/T.Rex cells. RNA samples were collected at the indicated time points following doxycycline induction and the PCR data were obtained from three independent RNA preparations from each time point, normalized to B2M mRNA and uninduced control samples. Error bars represent mean ± SD.

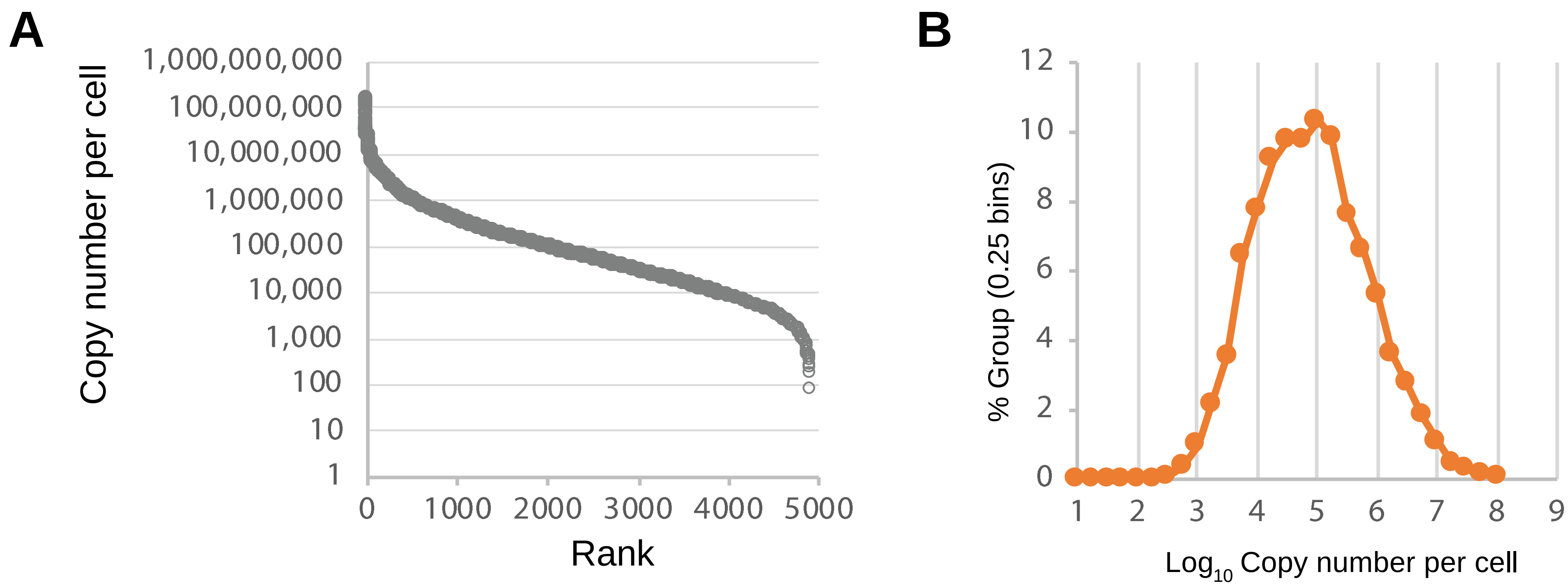

Figure S2. Absolute protein abundance dynamic range of the proteomics experiment to monitor changes in total cellular proteome in response to NEDP1 nanobody-RING expression. Related to Figure 5.

A: Ranked distribution of estimated protein copy number per cell (see materials and Methods) of 4907 proteins identified in the double-SILAC proteomics experiment shown in Figure 5. . B: Frequency distribution of log10 transformation of data shown in A.

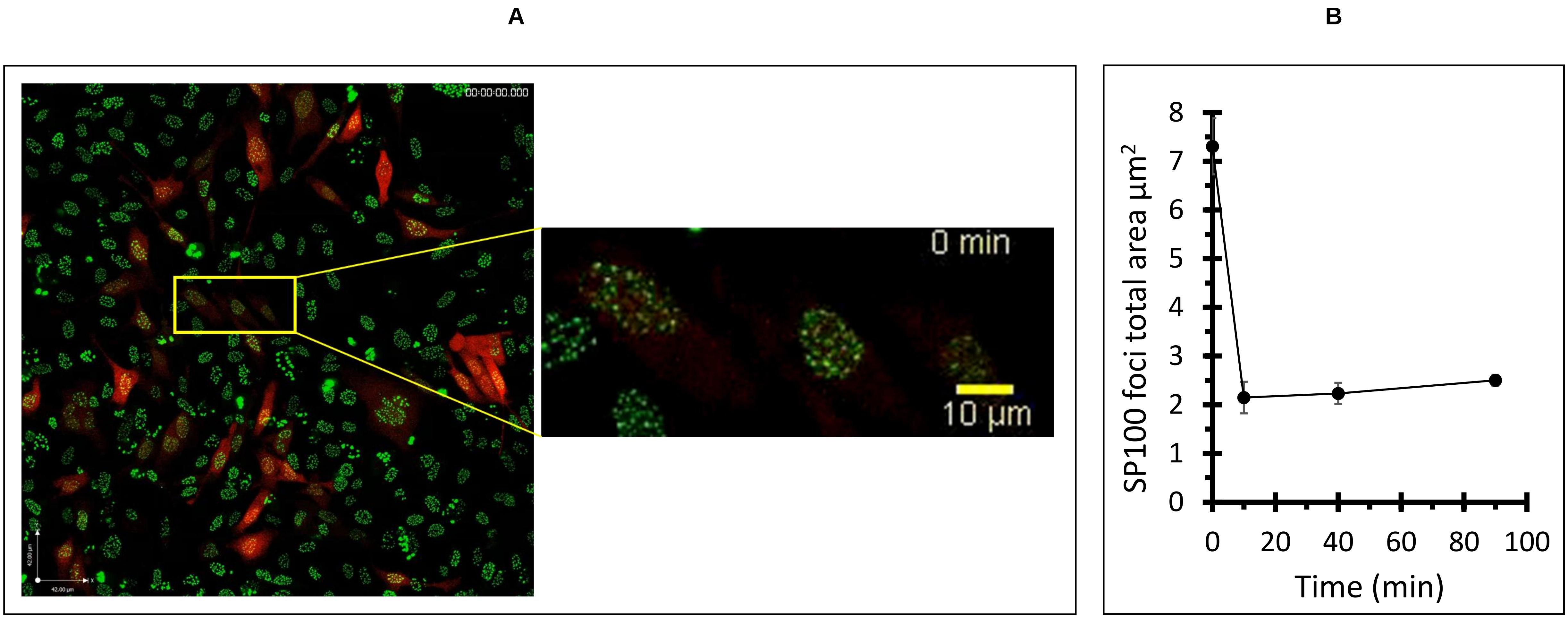

Fiure S3. Still of extended movie 1 to support figure 6D, ARMeD of YFP-PMLIII, and HC analysis of YFP-SP100 ARMeD. Related to Figure 6.

(A) Still of extended movie to support figure 5D by showing the entire field of injected cells and indicating the area magnified and used in the main figure 6D. (B) HC imaging of HEK293 YFP-SP100 cells electroporated with GNb-2xRING (1.5 pg/cell) and fixed at 10, 40 and 90 minutes. The total number of analysed cells was 12719 (control), 7745 (10 min), 8480 (40 min) and 14983 (90 min) and the plotted values represent the SP-100 foci total area/cell averaged from four wells ± SD.

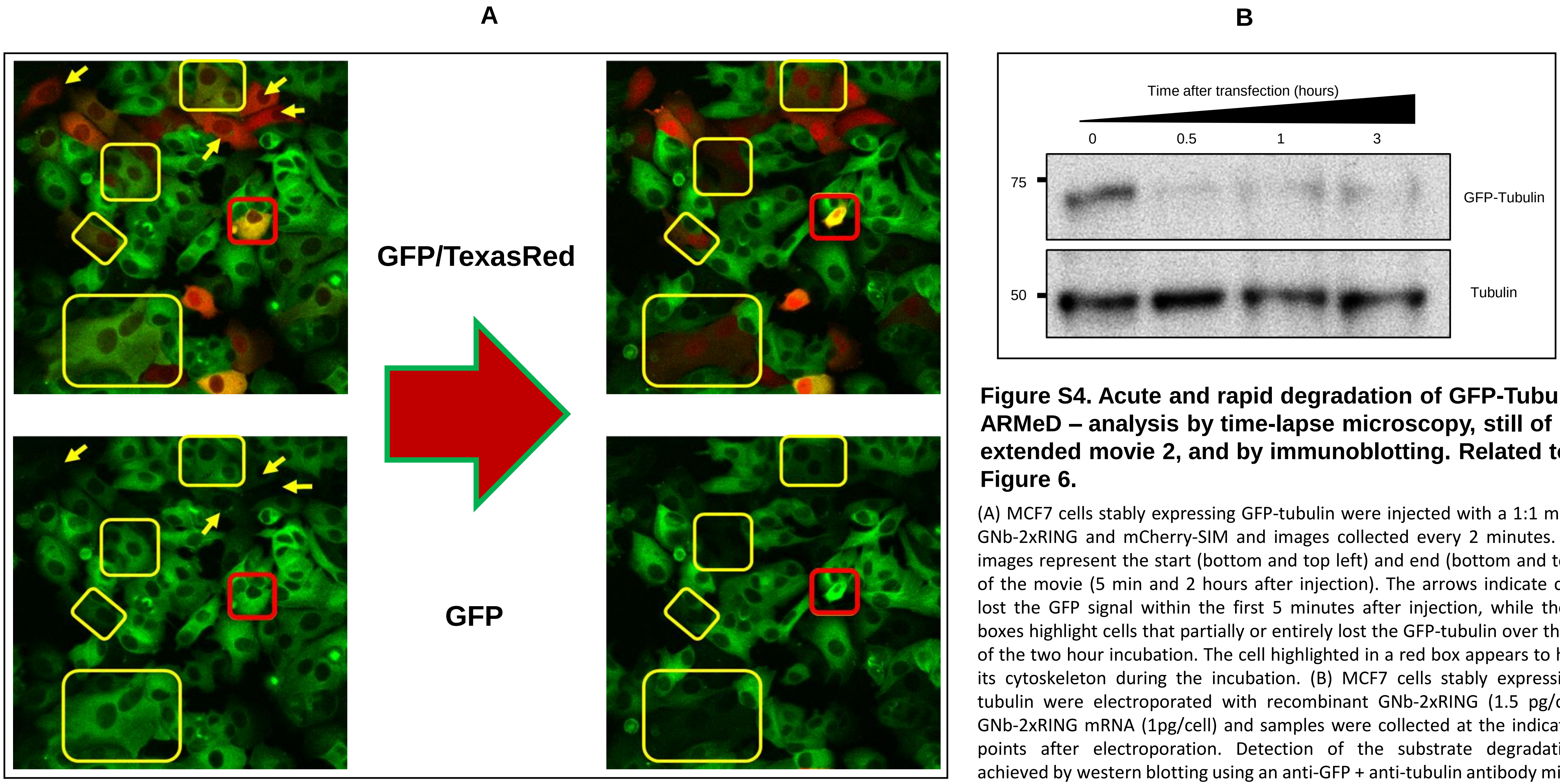

Figure S4. Acute and rapid degradation of GFP-Tubulin by ARMeD – analysis by time-lapse microscopy, still of extended movie 2, and by immunoblotting. Related to Figure 6.

(A) MCF7 cells stably expressing GFP-tubulin were injected with a 1:1 mixture of GNb-2xRING and mCherry-SIM and images collected every 2 minutes. The still images represent the start (bottom and top left) and end (bottom and top right) of the movie (5 min and 2 hours after injection). The arrows indicate cells that lost the GFP signal within the first 5 minutes after injection, while the yellow boxes highlight cells that partially or entirely lost the GFP-tubulin over the course of the two hour incubation. The cell highlighted in a red box appears to have lost its cytoskeleton during the incubation. (B) MCF7 cells stably expressing GFP-tubulin were electroporated with recombinant GNb-2xRING (1.5 pg/cell) and GNb-2xRING mRNA (1pg/cell) and samples were collected at the indicated time points after electroporation. Detection of the substrate degradation was achieved by western blotting using an anti-GFP + anti-tubulin antibody mix

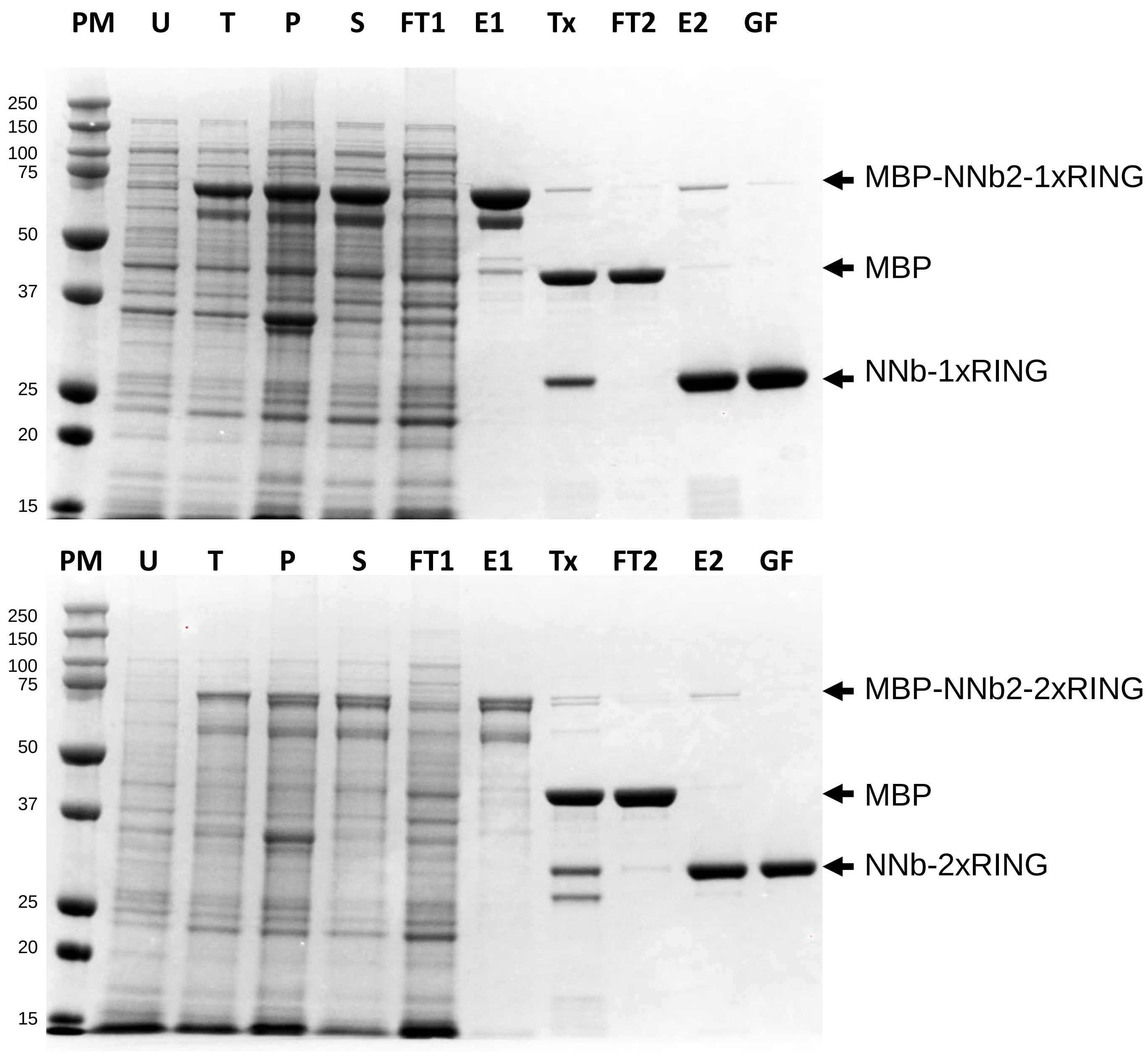

Figure S5. Purification of NEDP1 nanobody NNb2-1xRing and NNb2-2xRing fusion proteins. Related to Figure 7.

Purification of NEDP1 nanobody NNb2-1xRing and NNb2-2xRing fusion proteins: Coomassie-blue stained SDS-PAGE analysis of purified NEDP1 nanobody NNb2-1xRing (top) and NNb2-2xRing (bottom) fusion proteins. U: uninduced, T: induced total cell lysate, P: pellet, S: supernatant, Ft1: first nickel bead flow through, E1: nickel bead eluate, Tx: TEV protease cleaved, Ft2: SP column flow through, E2: SP column eluate, GF: gel filtrate.

A

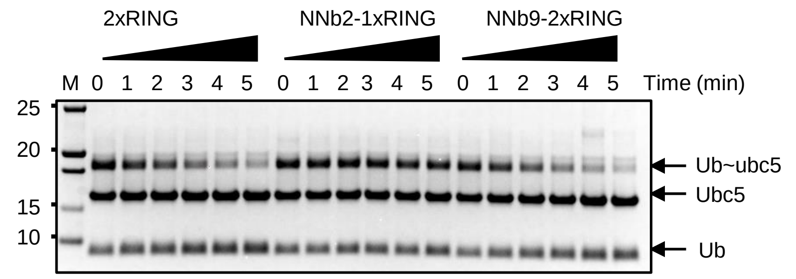

B

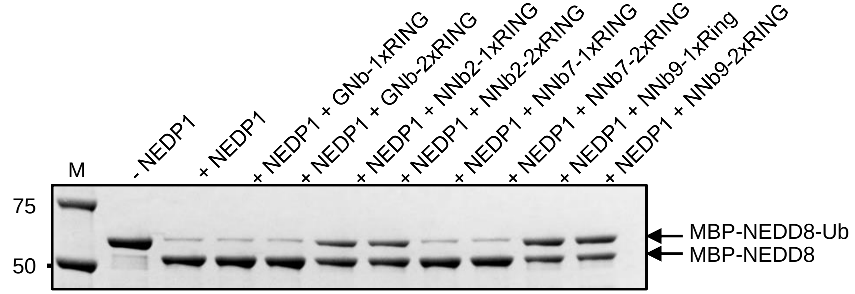

Figure S6. Comparative analysis of NEDP1 ARMeD fusions ubiquitination activity and NEDP1 inhibition. Related to Figure 7.

(A) For a direct comparison of the activity of the NNb2-1xRING and NNb9-2xRING in vitro they were tested by a Lysine discharge assay with ubiquitin loaded Ubc5 (Ub-Ubc5). A fused RNF4 RING (2xRING) served as a positive control. Samples were removed at the indicated times (minutes) and analysed by non-reducing SDS-PAGE. (B) MBP-NEDD8-Ubiquitin substrate was incubated without (- NEDP1) or with (+ NEDP1) 50 nM NEDP1 as inhibition negative and positive control, respectively. The reaction was incubated at 25°C for 15 min followed by SDS-PAGE and Coomassie staining for visualization. The ability of the shown nanobody-RING fusions to inhibit NEDP1 activity was then monitored in terms of their ability to prevent removal of ubiquitin from the substrate, leading to reduced accumulation of the cleaved product (lower band) and an increase of the uncleaved product when compared to the positive control (+NEDP1). GNb-1x and 2xRING served as non-specific nanobody negative control.

Table S3: DNA oligonucleotides used in the study. Related to STAR Methods.

Sequence (5'-3')	Used for
AAAAAAGCTAGCGAAGAAAGGAGAAGGCCAAGGAGAAATG	RNF4 RING PCR, forward. *Nhe*I site underlined.
AAAAAAGCGGCCGCTTATCATATATAAATGGGATGGTACCGTTTATGGTTG	RNF4 RING PCR, reverse. *Not*I site underlined
AAAAAACCATGGCCGATGTGCAGCTGGTTGAATC	GFP nanobody PCR, forward. *Nco*I site underlined.
AAAAAACCATGGCTCAGGTGCAGCTGCAAGAGAGCG	NEDP1 nanobody2/9 PCR, forward. *Nco*I site underlined.
AAAAAACCATGGCCCAGGTGCAGCTGCAG	NEDP1 nanobody7 PCR, forward. *Nco*I site underlined.
AAAAAATCTAGACTCGAGCGGCCGCTTATC	pCDNA5 FRT TO generic reverse PCR primer used for nanobody-1xRING/2xRING amplification
TAATACGACTCACTATAGGGAGA**GCCACCATG**GCCGATGTGCAGCTGGTTGAATC	GFP nanobody-RING in vitro transcription template forward amplification primer. T7 promoter + Kozak consensus (bold) underlined
TAATACGACTCACTATAGGGAGA**GCCACCATG**GTGAGCAAGGGCGAGGAGG	mCherry in vitro transcription template forward amplification primer. T7 promoter + Kozak consensus (bold) underlined
AAAAAAATTACTTGTACAGCTCGTCCATGCCGC	mCherry reverse amplification primer
AAAAAAGCTAGCGCTACCGGTCGCCACCATGGTGAGCAAGGGCGAGGAGC	EYFP amplification for creating pEFIRES-P-eYFP, forward primer. *Nhe*I and *Spe*I sites underlined.
AAAAAACTCGAGGCATGCACTAGTAGCGATCGCAGATCTGAGTCCGGACTTGTACAGCTCGTCCATGCCGAG	EYFP amplification for creating pEFIRES-P-eYFP, reverse primer. *Xho*I site underlined.
AAAAAAACTAGTATGAATGCGGGCCCCGGCTG	PARG forward amplification primer. *Spe*I site underlined.
AAAAAAGCGGCCGCTCAGGTCCCTGTCCTTTGCCCTG	PARG reverse amplification primer. *Not*I site underlined.
AGGCCGCTTTACCATTTCTC	GFP nanobody-RING qRT-PCR forward
AGATGAACTCCCTGAAACCAG	NDEDP1 nanobody2/9-RING qRT-PCR forward
CTTGGCCTTCTCCTTTCTTC	Generic reverse primer for nanobody-RING qRT-PCR
TCCAGCGTACTCCAAAGATTCAG	Beta-2 microglobulin (B2M) forward qRT-PCR primer
CAGTGGGGGTGAATTCAGTGT	Beta-2 microglobulin (B2M) reverse qRT-PCR primer
